# No benefit for elbow blocking on conservative treatment of distal radius fractures: A 6-month randomized controlled trial

**DOI:** 10.1371/journal.pone.0252667

**Published:** 2021-06-10

**Authors:** Aldo Okamura, Vinícius Ynoe de Moraes, Jorge Raduan Neto, Marcel Jun Tamaoki, Flavio Faloppa, João Carlos Belloti

**Affiliations:** 1 Department of Orthopaedics and Traumatology, Unit of Hand Surgery, Universidade Federal de São Paulo (Unifesp -EPM), São Paulo, Brazil; 2 Division of Orthopaedics and Traumatology, Hospital Municipal Dr. Fernando Mauro Pires da Rocha (Hospital do Campo Limpo), São Paulo, Brazil; 3 Hand Surgery Division, Hospital Alvorada Moema–United Health Group, São Paulo, Brazil; Monash University, AUSTRALIA

## Abstract

**Purpose:**

For displaced distal radius fracture, this trial aimed to compare an above-elbow (AE) and below-elbow (BE) cast at the end of a 24-week follow-up using the Disabilities of the Arm, Shoulder, and Hand (DASH) questionnaire as a primary outcome.

**Methods:**

This is a clinical trial with parallel groups (1:1) and a blinded evaluator. There are two non-surgical interventions: AE and BE. A total of 128 adult patients with acute (up to 7 days) displaced distal radius fracture of type A2-3, C1-3 by the AO classification were included. The follow-up was 24 weeks. The primary outcome was the DASH questionnaire at 24 weeks. Secondary outcomes were the maintenance of reduction by the evaluation of radiographic parameters, pain measured by VAS, PRWE, objective functional evaluation and rate of adverse effects.

**Results:**

The difference between the two groups in the DASH score at 24 weeks was not significant, with the mean (95% CI) DASH score being AE: 9.44 (2.70 to 16.17) vs. BE: 9.88 (3.19 to 16.57) (p = 0.895). The above-elbow group had a significantly greater worsening of the mean DASH score from baseline to 2 weeks (p < 0.001). No statistically significant differences were found between the 2 groups in any of the other follow-up assessments. Objective functional evaluation, PRWE, radiographical measures and rates of reduction loss were similar between groups. Above-elbow casting resulted in more adverse effects (mostly shoulder pain; 19 events vs. 9 events); RR = 0.39 (0.19–0.94); p = 0.033 at the end of six-month follow-up.

**Conclusions:**

This study did not demonstrate a difference between above-elbow and below-elbow cast in terms of DASH outcome at 6 months in non-surgical treatment of deviated distal radius fractures. However, below-elbow casting is less debilitating during the treatment period, has comparable performance in maintaining the reduction, and is related to fewer minor adverse effects than above-elbow casting.

## Introduction

Although distal radius fractures (DRF) are among the most frequent types of fracture of the upper limb [1], the best method of treatment and outcome of these fractures has not yet been fully defined [2,3]. Regarding non-surgical treatment, a Cochrane review based on randomized controlled trials has concluded that controversy remains in terms of the type of immobilization to be applied after the initial fracture reduction [4]. A recent overview of systematic reviews about the treatment of DRF in adults demonstrated that only two studies were on conservative treatment and none of them underwent meta-analysis [5]. A major systematic review on the topic was carried out by Handoll et al. which justified the absence of meta-analysis due to the low quality and heterogeneity in terms of interventions compared and outcome measurement of the included trials [4].

Additionally, there is no conclusive evidence of differences in outcome between different positions and methods of plaster and brace management for the common types of DRF [4,6–8].

Below-elbow (BE) casting is easier to apply, lighter, provides greater comfort, better function for daily life activities and possibly less articular stiffness of the elbow compared to AE cast [9–11]. Above-elbow casting (AE), which prevents the rotation of the forearm, may result in greater stability of the fracture and less risk of loss of reduction and need for re-reduction [12–14]. Other studies found similar results between immobilization methods in maintaining the initial fracture reduction [15–20].

We hypothesized that below-elbow cast participants would have the better patient-reported outcome (DASH) compared to above-elbow cast group at six-month follow-up.

## Method

This study was approved by the Local Research Ethics Committee under the number CAAE: 57857216.8.0000.5505 (UNIFESP) and CAAE:57857216.8.3001.5452 (Hospital Municipal Dr. Fernando Mauro Pires da Rocha) in Oct 2016. The protocol was under an identifier number–NCT03126175 (ClinicalTrials.gov), registered in April 2017. Publication of the protocol in March 2018 [21]. Patients were recruited between April 2018 and June 2019 and last follow-ups carried out on Dec 2019. No interim analysis was planned and conducted. This study followed the Consolidated Standards of Reporting Trials (CONSORT) reporting guideline. We made a few changes in the methods and outcomes after the protocol was published. These changes are described in more detail in the paper.

### Aim

This study aimed to compare above-elbow (AE) and below-elbow (BE) cast in the treatment of displaced distal radius fracture, at the end of a six-month follow-up using the DASH questionnaire as a primary outcome.

### Design and setting

Randomized controlled trial developed at Universidade Federal de São Paulo and Hospital Municipal Dr. Fernando Mauro Pires da Rocha, São Paulo, Brazil.

### Participant characteristics

Adults, both genders, with unilateral and closed acute displaced DRF, associated or not with ulnar styloid fractures with no other fractures, which may be closed reduced and met inclusion criteria.

### Inclusion criteria

Displaced and reducible fractures classified by AO as type A2, A3, C1, C2 and C3 were included if one of the following conditions was present:

- Radial height–loss > 2 mm [22–25]
- Radial Inclination—loss > 4° [24,27,28]
- Volar tilt > 10° dorsal angulation [6,25–27]
- Positive ulnar variance–loss > 3 mm [26–28]
- Intra-articular step off or gap > 2 mm [6,26–29]
- Carpal malalignment [30].

The contralateral side was used as a reference.

### Exclusion criteria

- Open fractures, bilateral fracture or associated with tendon or neurovascular lesions
- Time fracture > 7 days
- Associated carpal fractures
- Marginal fractures or fractures from shearing mechanism
- Irreducible fractures (closed method)
- Prior history of a degenerative or traumatic disorder of the affected or contralateral wrist joint
- Systemic disease or traumatic lesions associated with fracture that restrict the application of methods or the evaluation of results
- Cognitive deficit that does not allow the patient to understand the elements of the functional evaluation
- Consent form refusal.

### Radiological measurements

Palmar tilt, radial inclination, radial height, ulnar variance and intra-articular step off or gap were determined on posteroanterior (PA) and lateral (L) radiographs views obtained using a standardized procedure [31].

#### Initial treatment

All patients had a distal radius fracture upon arrival at the emergency room and had a clinical and radiographic examination, with bilateral x-rays of the wrist in PA and lateral views. On a pre-scheduled date (up to 7 days), the study participant was anesthetized and closed reduction of the fracture was performed under radioscopy to evaluate reducibility criteria. Patients with reducible fractures by a closed method were randomized and treated by one of the two methods of the study. Patients without a reducible distal radius fracture by closed method were excluded from the study and have received surgical treatment on a date to be scheduled. The details can be found elsewhere [21].

#### Anesthesia

Intravenous anesthesia was performed by an aseptic technique with an anesthesiologist in the operating room. A simple bolus injection with propofol (infusion rate 180 mcg.kg^-1^.min^-1^) in combination with an opioid (fentanyl 5–10 mcg.kg^-1^) was adjusted to the individual needs of each patient and repeated as many times as necessary according to the anesthesiologist’s criteria [32,33]. The purpose of general anesthesia was to enable the best reduction possible and maximum comfort to the patient.

#### Method for closed reduction and immobilization

The patient was submitted to the closed reduction of the fracture through a traction and counter-traction technique under radioscopy control. Materials for application of the two cast techniques were available in the operating room. Initially, all patients received a short radial cast that was performed with a 20 cm wide plaster of Paris bandage cut to fit the thumb. The cast was applied to the radial aspect of the wrist covering the volar and dorsal portion of the radius to the elbow. The cast was molded with three-point fixation under radioscopy control [34]. Patients randomized to the AE cast received a complementation of immobilization with a 15 cm width cast on the ulnar aspect of the forearm that begins at the middle of the forearm and extends into the armpit. We conducted the elbow splint (AE group) carefully, so it does not exceed half of the forearm, avoiding its appearance on the wrist radiographs and preserving the security of the allocation at the time of the assessment. The elbow was immobilized at 90 degrees, in a neutral position to block pronosupination. Regardless of the immobilization adopted, all wrists were positioned with slight flexion and ulnar deviation. Patients were encouraged to actively move their fingers and the ipsilateral shoulder. Patients with above-elbow immobilization remained for 4 weeks with the cast followed by 2 weeks of below-elbow immobilization. The immobilization was removed after 6 weeks.

#### Clinical outcomes

The primary outcome was changed during the recruitment phase of the trial based on the initiative of the Core Outcomes Measures in Effectiveness Trials (COMET) [35]. We decided to adopt DASH at 24 weeks as the only primary outcome to increase the homogeneity of clinical trials on this topic. The calculation of the sample in this study was based on the clinically significant difference in DASH scores. Radiographic parameters were considered as a secondary outcome.

The outcome assessors were not directly involved in the study (orthopedic residents). They assisted patients in completing the self-reported questionnaires, measured pain (VAS), palmar grip, joint range of motion, radiographic indices and recorded adverse effects of the treatment. To blind these assessors to the outcomes at 1, 2, 3, 4 and 6 weeks (before cast removal) all participants were asked to use appropriate clothing (long sleeve) and identical large velpeau shoulder immobilizer that covered most of the upper limb. The participants were instructed not to reveal the treatment that they had undergone. For the outcomes at 6, 8, 12 and 24 weeks (after cast removal) the assessors were blinded to the patient assignment groups. The data given to the statistician contained only numbers without revealing the group allocation. It was not possible to blind the participants.

#### Primary outcome

Functional status was evaluated by means of Disabilities of the Arm, Shoulder, and Hand Questionnaire (DASH) completed at 24 weeks to assess upper limb disability.

The DASH is a patient-reported outcome instrument developed to measure upper extremity disability and symptoms, resulting in a score ranging from no disability (0) to most severe disability (100). Questionnaires with unanswered responses were analyzed by the standards of the user’s manual [36].

#### Secondary outcomes

Maintenance of reduction by evaluation of the wrist in PA and lateral radiographs at the following intervals: 0, 1, 2, 3, 4, 6, 8, 12 and 24 weeks after fracture reduction. The radial height, radial inclination, palmar tilt, ulnar variance, intra-articular step off or gap and carpal alignment were used to determine maintenance of reduction at every follow-up visit. We considered maintenance of reduction if there was:

- loss of reduction ≤ 2 mm in radial height
- loss of reduction ≤ 4 degrees in radial inclination
- dorsal angulation ≤ 10°
- ≤ 2 mm intra-articular step off
- positive ulnar variance ≤ 3 mm
- any carpal malalignment.

The contralateral side was used as a reference.

After immobilization in the operating room and at each outpatient return, participants were given radiographs and were subsequently seen by two assistants not directly related to the study (orthopedics residents) who evaluated whether or not there was a loss of reduction by defined radiographic criteria. The assistants were blinded to the treatment group at the time of the assessment. Loss of reduction assessed as a dichotomous variable (yes or no), were recorded with their date of occurrence and method of treatment. Disagreements were resolved by the principal investigator (AO). Thus, an x-ray exam and radiographic measurements were taken immediately after plaster immobilization with the patient still in the operating room. Radiographic measurements were made at a single time and not on different occasions as described in the protocol. These modifications facilitated a quick analysis of the initial post-reduction status for decision-making regarding the maintenance of conservative treatment.

In cases where there is loss of reduction, patients were informed and surgical treatment indicated by surgeons in accordance with patient preferences. Otherwise, patients underwent a six-week cast immobilization period. Guided physiotherapy was introduced if needed. Patients who developed such complications were clinically followed and the results were included in their originally allocated group, according to the intention-to-treat principle (ITT).

The PRWE scores was obtained at 8, 12, and 24 weeks [37]. Pain in the wrist, elbow and shoulder was measured separately in all visits at 1, 2, 3, 4, 6, 8, 12 and 24 weeks after fracture reduction by VAS [38,39]. Range of motion was measured for the wrist, and a goniometer was employed to measure wrist flexion, extension, ulnar deviation, radial deviation and pronosupination at the 6, 8, 12 and 24-weeks follow-up visits. Flexion–extension of the elbow was measured at the 6, 8, 12 and 24-weeks follow-up visit. Palmar grip strength was assessed with a digital dynamometer at the 8, 12 and 24-weeks follow-up visit.

#### Adverse effects

Any clinical situation requiring treatment (clinical or surgical procedure), not provided in the protocol, was considered a complication and stratified into major and minor. Minor complications were those that resolve without specific treatment. Major complications required occupational therapy, steroid injections, additional immobilization or protocol changes.

#### Statistical analysis

We performed a preliminary analysis with the t-test to assess the primary outcome to justify the sample size (S1 Appendix). The 95% confidence interval (CI) were calculated for the differences between the selected outcome measures. Baseline univariate between-group tests was done to compare groups on outcome variables, clinical and demographic data. Data were analysed following “intention-to-treat” principles. Linear mixed effects modelling was used to test differences between groups in continuous outcome measures, namely DASH, PRWE, pain VAS pain scores, range of motion, grip strength and radiographic variables. Overall effects of time and time by group interactions were tested by likelihood ratio chi-squared tests. To perform all statistical analysis in the presence of missing data, we imputed the missing data using multivariate normal imputation with MCMC algorithm. Ten imputations were performed. Finally, the results were pooled utilizing the Rubin’s rules [40,41]. For binary outcomes (complication and reduction loss) the relative risk (RR) and the absolute risk were reported. We made an additional analysis not prespecified in the trial protocol using the Chi-Square test to measure the relationship between "Reduction Loss" with "Age". The rationale behind these analyses is that patient age has been most consistently a significant predictor for loss of alignment [42]. These additional analyses are identified and described in the appendix file (S1 Appendix). The data were analyzed using SPSS 20.0 and STATA 12.

#### Randomization

Permuted block randomization with block size 8 was performed using randomization software (www.randomizer.org) [43]. The allocation (1:1 ratio fashion) of patients in the AE or BE groups were performed using opaque envelopes numbered on the outside with consecutive numbers. Additionally, the envelope was only opened in the operating room after verification of fracture reducibility. In order to avoid undesirable change on patient allocation and before envelope opening, the patients’ name was written on the upper-front side of the envelope. This procedure was delegated to a person who was not directly connected to the study (orthopedic residents). Investigators were blinded to the size of block.

#### Sample size

The sample size was based on data derived from one recent randomized clinical trial [17]. We considered as clinically relevant differences in DASH scores when scores were greater than 10 points and a standard deviation of 15 points [44], calculations were based on two-sided Student’s t-test (alpha 0.05), a study with a statistical power of 95% was chosen resulting in a 58-patient sample size per group. We added an extra 10% to balance after follow-up losses. Thus, our inclusion target was 64 patients per group. Minitab 16 was used for sample size calculation.

## Results

**Fig 1:**
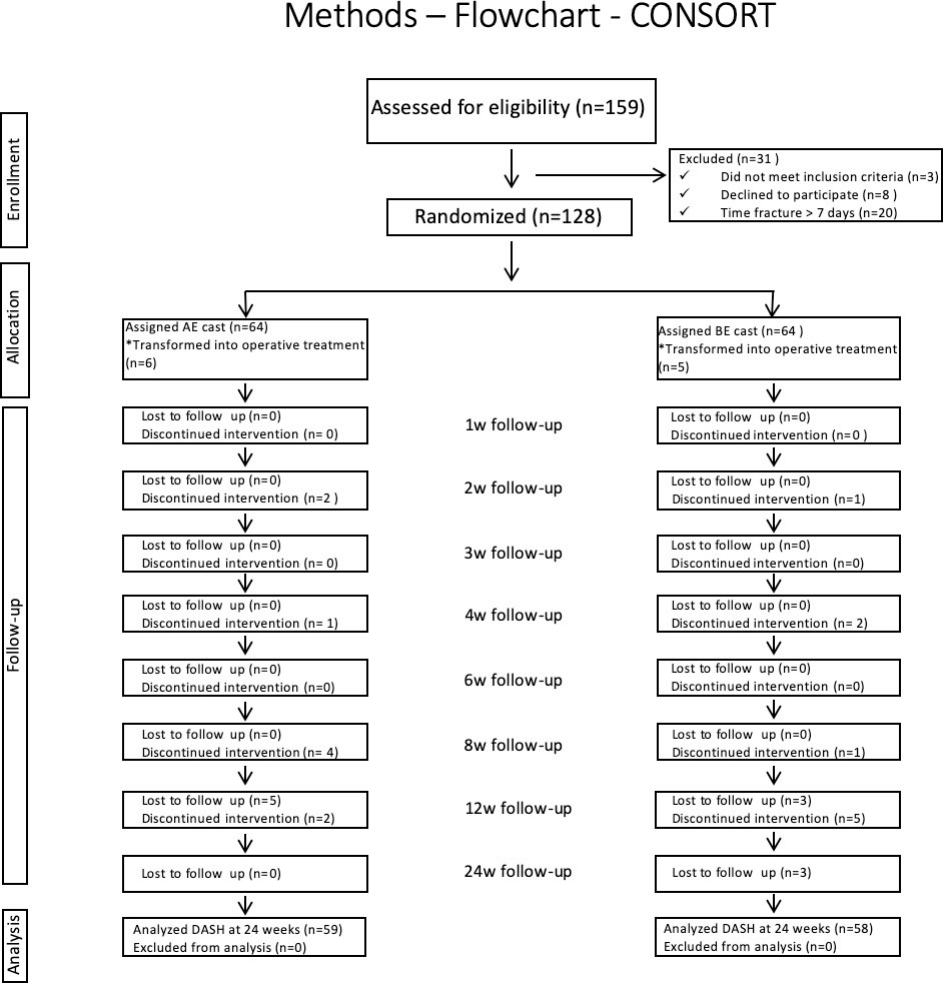
Flowchart according to CONSORT.

From the 128 included patients, 117 (AE: 59 and BE: 58) patients were available for assessment in the 24-week follow-up. Operative treatment due to loss of reduction of fracture was performed for six patients (10%) in the AE group and for five patients (8.6%) in BE cast group as described in CONSORT (Fig 1). Follow-up losses were balanced between groups (AE: 5 losses; BE: 6 losses). These losses were all concentrated in the 12-week (8 losses) and 24-week (3 losses) follow up. Discontinued interventions were balanced between groups (AE: 9; BE: 9).

Most of our sample was composed of elderly adults and fractures were a result of low-energy trauma (87 patients; 74.3%). Additional baseline characteristics demonstrated balanced groups in Table 1.

**Table 1:** Baseline characteristics of patients in the AE and BE groups.

Variable	AE (N = 64)	BE (N = 64)
Age, mean (SD)	62.97 (13.03)	60.52 (14.74)
Sex, female, n (%)	47 (73.4)	41 (64.1)
Time between fracture and reduction, days, mean (SD)	3.5 (2.6)	3.9 (2.2)
Fracture, right side, n (%)	31 (48.4)	36 (56.3)
Handed side, right, n (%)	62 (96.9)	63 (98.4)
AO classification type, n (%)		
• A2	11(17.2)	16 (25)
• A3	26 (40.6)	29 (45.3)
• C1	9 (14.1)	5 (7.8)
• C2	16 (25)	13 (20.3)
• C3	2 (3.1)	1 (16.6)

### Patient-reported functional assessment

The primary outcome DASH score was measured at 24 weeks. The mean (95% CI) DASH score was 9.44 (2.70 to 16.17) for AE and 9.88 (3.19 to 16.57) for BE (p = 0.895). The above-elbow group had a significantly greater worsening of the mean DASH score from baseline to 2 weeks (p < 0.001) (Table 2). No statistically significant differences were found between the two groups in any of the other follow-up assessments. The differences in the mean change in DASH over time were statistically significant (p < 0.001). For both groups, DASH improved from 2 to 6 weeks, 6 to 8 weeks, and 8 to 12 weeks, and for AE but not the BE group, DASH also improved from 12 to 24 weeks.

**Table 2:** Results of patient-reported outcome (DASH), show as mean (95%CI).

	Group		P- value	P- value^a^
	AE (N = 64)	BE (N = 64)		
2 weeks	70.4 (63.63 to 77.16)	45.01 (38.34 to 51.68)	<0.001	<0.001
6 weeks	37.12 (30.53 to 43.72)	34.28 (27.68 to 40.87)	0.392	1.000
8 weeks	24.98 (18.37 to 31.6)	24.35 (17.74 to 30.97)	0.850	1.000
12 weeks	15.12 (8.37 to 21.88)	17.26 (10.35 to 24.18)	0.525	1.000
24 weeks	9.44 (2.70 to 16.17)	9.88 (3.19 to 16.57)	0.895	1.000

Mixed Linear Model—Group effect (p<0.001); Time effect (p<0.001); interaction Group x Time (p < 0.001).

P Value—Multiple Comparisons using contrasts without and with Bonferroni correction (^a^).

No statistically significant differences were found in the mean PRWE scores or changes in the mean score over time between 2 groups in any follow-up assessment (Table 3). For both groups, the mean PRWE score decreased over time; PRWE: 8W > 12 W > 24W (p < 0.001).

**Table 3:** Results of PRWE and GRIP strength, show as mean (95%CI).

	Group		P-value
	AE (N = 64)	BE (N = 64)	Group x Time
**PRWE**			0.848
8 weeks	26.00 (21.08 to 30.92)	25.71 (20.79 to 30.63)	
12 weeks	15.35 (10.30 to 20.40)	16.5 (11.46 to 21.53)	
24 weeks	7.36 (2.38 to 12.33)	9.18 (4.19 to 14.18)	
**GRIP**			0.933
8 weeks	8.04 (5.95 to 10.13)	8.78 (6.69 to 10.87)	
12 weeks	12.09 (9.97 to 14.2)	13.42 (11.3 to 15.53)	
24 weeks	17.45 (15.33 to 19.57)	18.48 (16.35 to 20.61)	

P-value–Mixed Linear Model.

### Assessment of fracture reduction losses

Nearly half of the patients had a loss of the achieved reduction (from the tight a priori defined criteria), with most occurring until week 3 (63/69; 91.3%). The rate of reduction loss was similar between above-elbow and below-elbow casting; AE: 35 (54.7%) vs. BE: 34 (53.1%); RR = 0.94 (0.71–1.34); p = 0.859. The reduction loss was greater in the population over 60 years old (76.8%); RR = 0.46 (0.31 to 0.66); p < 0.001 (S1 Appendix).

### Pain

Differences in the wrist, elbow and shoulder pain, as measured by VAS, were not statistically significant between the 2 groups at any follow-up assessment, although pain levels in the AE group were slightly higher. At 24-weeks, the mean (95% CI) wrist VAS was 7.03 (-0.13 to 14.19) for AE and 4.89 (-2.43 to 12.21) for BE; and this difference was not statistically significant, p = 0.787. The mean elbow VAS was 0.88 (-2.56 to 4.31) for AE and 0 (-3.50 to 3.41) for BE, and this difference was also non-significant, p = 0.396. The mean shoulder VAS was 3.52 (-2.20 to 9.24) for AE and 2.69 (-3.10 to 8.49) for BE; again, the difference was non-significant, p = 0.361 (Table 4). Only wrist pain showed a significant change over time for both groups (wrist VAS: 1w > 2w > 3w > 4w > 6w > 8w > 12 w > 24w (p < 0.001).

**Table 4:** Results of pain in wrist, elbow and shoulder by visual analog scale (VAS), show as mean (95%CI).

Visual analog scale (VAS)	Group		P-Value
	AE (N = 64)	BE (N = 64)	Group x Time
**Wrist**			0.787
1 week	31.08 (24.00 to 38.16)	28.02 (20.94 to 35.09)	
2 weeks	24.58 (17.40 to 31.76)	20.1 (12.95 to 27.24)	
3 weeks	22.42 (15.34 to 29.50)	14.44 (7.36 to 21.52)	
4 weeks	15.46 (8.34 to 22.57)	14.04 (6.89 to 21.19)	
6 weeks	17.67 (10.59 to 24.75)	12.24 (5.16 to 19.32)	
8 weeks	19.95 (12.69 to 27.2)	13.28 (6.16 to 20.39)	
12 weeks	16.01 (8.54 to 23.48)	15.17 (7.84 to 22.51)	
24 weeks	7.03 (-0.13 to 14.19)	4.89 (-2.43 to 12.21)	
**Elbow**			0.396
1 week	4.69 (1.27 to 8.11)	1.94 (-1.48 to 5.36)	
2 weeks	5.46 (2.00 to 8.92)	2.10 (-1.34 to 5.55)	
3 weeks	7.02 (3.60 to 10.43)	2.17 (-1.25 to 5.59)	
4 weeks	4.05 (0.61 to 7.48)	1.75 (-1.70 to 5.20)	
6 weeks	3.77 (0.35 to 7.18)	1.34 (-2.08 to 4.76)	
8 weeks	3.70 (0.24 to 7.15)	1.69 (-1.74 to 5.12)	
12 weeks	1.94 (-1.66 to 5.54)	2.84 (-0.71 to 6.38)	
24 weeks	0.88 (-2.56 to 4.31)	0.00 (-3.50 to 3.41)	
**Shoulder**			0.361
1 week	8.03 (2.39 to 13.67)	3.67 (-1.97 to 9.31)	
2 weeks	9.39 (3.69 to 15.10)	6.61 (0.93 to 12.29)	
3 weeks	13.08 (7.44 to 18.72)	5.94 (0.30 to 11.58)	
4 weeks	14.56 (8.90 to 20.23)	5.67 (-0.03 to 11.36)	
6 weeks	8.72 (3.08 to 14.36)	6.11 (0.47 to 11.75)	
8 weeks	8.86 (3.15 to 14.57)	5.78 (0.12 to 11.44)	
12 weeks	6.93 (1.06 to 12.80)	5.96 (0.19 to 11.73)	
24 weeks	3.52 (-2.20 to 9.24)	2.69 (-3.10 to 8.49)	

P-value—Mixed Linear Model.

### Radiographical assessments

No significant differences were found in radial height (RH), palmar tilt (PT), and intra-articular step (ST) between groups (Table 5). For both groups, the differences in radiographic variables (RH, RI, PT, UV) over the time were statistically significant (p < 0.001). In multiple comparisons using contrast with Bonferroni correction (both groups), we found that after CR > 1W = 2W = 3W = 4W = 6W = 8W = 12W = 24W > before CR for RH, RI and PT. For UV we found that before CR > after CR = 1W = 2W = 3W = 4W = 6W = 8W = 12W = 24W.

**Table 5:** Results of radiographic assessment, show as mean (95%CI).

Radiographical measures	Group		P-value
	AE (N = 64)	BE (N = 64)	Group x Time
**Radial height (mm)**			1.000
Before CR	5.84 (4.82 to 6.87)	6.13 (5.10 to 7.15)	
After CR	10.03 (9.00 to 11.06)	10.55 (9.52 to 11.58)	
1 week	8.89 (7.86 to 9.92)	9.34 (8.32 to 10.37)	
2 weeks	8.36 (7.33 to 9.39)	8.87 (7.84 to 9.90)	
3 weeks	7.88 (6.85 to 8.90)	8.53 (7.50 to 9.56)	
4 weeks	7.77 (6.74 to 8.80)	8.31 (7.28 to 9.34)	
6 weeks	7.56 (6.53 to 8.59)	8.13 (7.10 to 9.15)	
8 weeks	7.55 (6.52 to 8.58)	8.01 (6.99 to 9.04)	
12 weeks	7.50 (6.47 to 8.53)	7.94 (6.91 to 8.97)	
24 weeks	7.49 (6.46 to 8.52)	7.89 (6.86 to 8.92)	
**Radial inclination (**^**°**^**)**			0.999
Before CR	13.33 (11.75 to 14.90)	14.78 (13.20 to 16.36)	
After CR	19.69 (18.11 to 21.26)	20.88 (19.30 to 22.45)	
1 week	18.14 (16.56 to 19.72)	19.25 (17.67 to 20.83)	
2 weeks	17.21 (15.63 to 18.79)	18.80 (17.22 to 20.38)	
3 weeks	16.61 (15.03 to 18.19)	18.45 (16.88 to 20.03)	
4 weeks	16.62 (15.04 to 18.20)	18.40 (16.82 to 19.98)	
6 weeks	16.38 (14.80 to 17.95)	18.27 (16.69 to 19.84)	
8 weeks	16.29 (14.71 to 17.87)	17.91 (16.33 to 19.49)	
12 weeks	16.30 (14.73 to 17.88)	18.05 (16.47 to 19.63)	
24 weeks	16.31 (14.73 to 17.89)	18.00 (16.42 to 19.58)	
**Palmar tilt (**^**o**^**)**			0.992
Before CR	-18.27 (-21.46 to -15.07)	-18.81 (-22.01 to -15.61)	
After CR	5.61 (2.41 to 8.81)	7.05 (3.85 to 10.24)	
1 week	2.91 (-0.29 to 6.10)	4.59 (1.40 to 7.79)	
2 weeks	1.28 (-1.92 to 4.49)	2.70 (-0.50 to 5.90)	
3 weeks	-0.42 (-3.62 to 2.78)	1.23 (-1.96 to 4.43)	
4 weeks	-0.47 (-3.67 to 2.73)	0.22 (-2.98 to 3.42)	
6 weeks	-1.03 (-4.23 to 2.17)	-0.30 (-3.49 to 2.90)	
8 weeks	-1.25 (-4.45 to 1.94)	-0.60 (-3.80 to 2.60)	
12 weeks	-1.33 (-4.53 to 1.86)	-0.72 (-3.91 to 2.48)	
24 weeks	-1.27 (-4.47 to 1.93)	-1.04 (-4.25 to 2.16)	
**Ulnar variance (mm)**			0.961
Before CR	2.33 (1.58 to 3.08)	1.31 (0.56 to 2.06)	
After CR	0.59 (-0.16 to 1.35)	0.33 (-0.42 to 1.08)	
1 week	0.86 (0.11 to 1.61)	0.59 (-0.16 to 1.35)	
2 weeks	1.22 (0.47 to 1.98)	0.73 (-0.02 to 1.48)	
3 weeks	1.58 (0.83 to 2.33)	1.05 (0.29 to 1.80)	
4 weeks	1.81 (1.05 to 2.56)	1.27 (0.51 to 2.02)	
6 weeks	1.94 (1.19 to 2.69)	1.45 (0.70 to 2.21)	
8 weeks	1.94 (1.18 to 2.69)	1.47 (0.72 to 2.22)	
12 weeks	1.95 (1.19 to 2.70)	1.37 (0.61 to 2.12)	
24 weeks	1.93 (1.18 to 2.68)	1.41 (0.66 to 2.17)	
**Articular step (mm)**			0.909
Before CR	0.53 (0.32 to 0.74)	0.34 (0.13 to 0.56)	
After CR	0.05 (-0.17 to 0.26)	0.03 (-0.18 to 0.24)	
1 week	0.13 (-0.09 to 0.34)	0.09 (-0.12 to 0.31)	
2 weeks	0.13 (-0.09 to 0.34)	0.09 (-0.12 to 0.31)	
3 weeks	0.13 (-0.09 to 0.34)	0.08 (-0.13 to 0.29)	
4 weeks	0.13 (-0.09 to 0.34)	0.11 (-0.10 to 0.32)	
6 weeks	0.13 (-0.09 to 0.34)	0.16 (-0.06 to 0.37)	
8 weeks	0.13 (-0.09 to 0.34)	0.16 (-0.06 to 0.37)	
12 weeks	0.14 (-0.08 to 0.36)	0.18 (-0.04 to 0.39)	
24 weeks	0.13 (-0.09 to 0.34)	0.16 (-0.06 to 0.37)	

P-value—Mixed Linear Model.

CR = closed reduction.

### Strength and range of motion

The mean (95% CI) grip strength, measured by dynamometry at 24-weeks, was 17.45 (15.33 to 19.57) for AE and 18.48 (16.35 to 20.61) for BE, with a non-significant difference, p = 0.933 (Table 3). The differences in grip strength between the two groups and the differences in the mean change in grip strength over the time were not statistically significant. For both groups, grip strength increased over time (GRIP: 8W < 12W < 24W; p < 0.001).

Elbow motion at 24-weeks was similar between groups. The mean (95% CI) elbow flexion was 148.44 (147.61 to 149.26) for AE and 148.36 (147.53 to 149.18) for BE, with a non-significant difference, p = 0.999, and the mean extension was -2.86 (-4.46 to -1.06) for AE and -3.20 (-5.01 to -1.4) for BE, with a non-significant difference, p = 0.999, at 24-weeks. The mean (95% CI) wrist range-of-motion at 24-week was assessed in terms of pronation, AE: 87.53 (83.06 to 92.01), BE: 86.99 (82.51 to 91.47); p = 0.278; supination: AE: 85.33 (79.03 to 91.63), BE: 83.45 (77.12 to 89.77); p = 0.725; wrist flexion: AE: 55.12 (51.54 to 58.71), BE: 53.62 (50.01 to 57.23), p = 0.783; wrist extension: AE: 61.84 (56.91 to 66.78), BE: 60.38 (55.40 to 65.36), p = 0.750; radial deviation: AE: 22.72 (19.83 to 25.61), BE: 22.25 (19.35 to 25.15), p = 0.878; ulnar deviation: AE: 35.37 (32.37 to 38.38), BE: 35.66 (32.64 to 38.69), p = 0.740. No statistically significant differences between the groups were found in any range of motion variable (Table 6). For both groups, wrist flexion (WF), extension (WE), radial deviation (RD), ulnar deviation (UD) and forearm pronation (P) and supination (S) improved significantly over time (WF, WE, RD, UD, P, S: 6W < 8W < 12W < 24W; except for RD: 12W = 24W; p < 0.001).

**Table 6:** Results of physical examination, show as mean (95%CI).

Range of motion	Group		P-value
	AE (N = 64)	BE (N = 64)	Group x Time
**Wrist flexion**			0.783
6 weeks	39.05 (35.52 to 42.58)	37.63 (34.09 to 41.16)	
8 weeks	47.59 (44.02 to 51.16)	43.85 (40.31 to 47.39)	
12 weeks	51.58 (47.95 to 55.21)	48.84 (45.27 to 52.42)	
24 weeks	55.12 (51.54 to 58.71)	53.62 (50.01 to 57.23)	
**Wrist extension**			0.750
6 weeks	26.89 (22.03 to 31.76)	28.38 (23.51 to 33.24)	
8 weeks	43.81 (38.89 to 48.73)	41.54 (36.66 to 46.42)	
12 weeks	54.26 (49.30 to 59.23)	53.30 (48.39 to 58.21)	
24 weeks	61.84 (56.91 to 66.78)	60.38 (55.40 to 65.36)	
**Radial deviation**			0.878
6 weeks	13.47 (10.64 to 16.3)	11.80 (8.97 to 14.63)	
8 weeks	18.26 (15.39 to 21.12)	16.34 (13.5 to 19.18)	
12 weeks	21.19 (18.30 to 24.09)	20.45 (17.59 to 23.32)	
24 weeks	22.72 (19.83 to 25.61)	22.25 (19.35 to 25.15)	
**Ulnar deviation**			0.740
6 weeks	22.19 (19.23 to 25.15)	24.45 (21.49 to 27.41)	
8 weeks	28.83 (25.84 to 31.82)	29.37 (26.41 to 32.34)	
12 weeks	32.76 (29.75 to 35.78)	32.84 (29.86 to 35.82)	
24 weeks	35.37 (32.37 to 38.38)	35.66 (32.64 to 38.69)	
**Pronation**			0.278
6 weeks	64.42 (59.96 to 68.88)	69.92 (65.46 to 74.38)	
8 weeks	75.97 (71.47 to 80.46)	78.55 (74.09 to 83.02)	
12 weeks	83.54 (79.04 to 88.04)	84.27 (79.79 to 88.75)	
24 weeks	87.53 (83.06 to 92.01)	86.99 (82.51 to 91.47)	
**Supination**			0.725
6 weeks	48.58 (42.31 to 54.85)	46.59 (40.33 to 52.86)	
8 weeks	68.14 (61.82 to 74.47)	61.66 (55.37 to 67.94)	
12 weeks	79.96 (73.57 to 86.35)	76.00 (69.65 to 82.36)	
24 weeks	85.33 (79.03 to 91.63)	83.45 (77.12 to 89.77)	
**Elbow flexion**			0.999
6 weeks	148.44 (147.61 to 149.26)	148.28 (147.46 to 149.11)	
8 weeks	148.44 (147.62 to 149.26)	148.36 (147.53 to 149.18)	
12 weeks	148.43 (147.61 to 149.26)	148.30 (147.46 to 149.14)	
24 weeks	148.44 (147.61 to 149.26)	148.36 (147.53 to 149.18)	
**Elbow extension**			0.999
6 weeks	-2.66 (-4.46 to -0.85)	-3.05 (-4.85 to -1.24)	
8 weeks	-2.35 (-4.15 to -0.55)	-3.05 (-4.85 to -1.24)	
12 weeks	-2.96 (-4.76 to -1.15)	-3.13 (-4.93 to -1.32)	
24 weeks	-2.86 (-4.46 to -1.06)	-3.20 (-5.01 to -1.40)	

### Adverse effects and malunion

Most of the complications were shoulder pain, 9 participants in the BE cast group and 17 participants in the AE cast group had ipsilateral shoulder pain greater than 20 points (a clinically significant difference) in VAS for more than 3 consecutive visits. Complex regional pain syndrome (AE: 1 patient); DRUJ instability (AE: 1 patient). When grouped, complications were higher in the above-the-elbow cast group (9 events vs. 19 events); RR = 0.39 (0.19–0.94); p = 0.033. We found high rates of malunion; AE: 29 (45.3%) vs. BE: 29 (45.3%); RR = 1.00 (0.50–2.01); p = 1.00. Symptomatic patients were referred to physiotherapy and maintained with medical supervision.

## Discussion

The aphorisma for elbow blocking dates from Sarmiento’s 1975 study and may have many advocates [13,14]. However, this finding may not be true, as some trials have consistently demonstrated that the biomechanical plausibility of his reasoning may not translate into clinical practice [17–20]. The present study provides Level-I evidence concerning non-surgical treatment of deviated distal radius fractures and is the first randomized clinical trial (RCT) comparing above-elbow (AE) and below-elbow (BE) cast that uses DASH as the primary outcome throughout treatment.

In contrast to our initial hypothesis, we could not identify a difference between the groups with regard to upper limb function measured with the DASH questionnaire at a 24-week follow-up. However, the below-elbow (BE) group had a statistically more favorable mean DASH score at 2 weeks (AE:70.4 vs. BE: 45.01, p < 0.001). Only three randomized clinical trials (RCTs) comparing conservative treatment techniques for distal radius fractures used the DASH self-assessment questionnaire [15,17,19]. This is the only RCT that uses DASH as the primary outcome throughout treatment. BONG et al. used short-term follow-up data (7 to 10 days after the injury) [15]. Therefore, it is not a reliable tool to compare immobilization methods. PARK et al. applied the DASH questionnaire in the initial assessment, and at the 3rd and 6th month after reduction. For the initial assessment, according to the author, all participants used the same immobilization (sugar tong), which was replaced in this view. In the 3rd and 6th months, both treatment groups were already without immobilization and not measuring the impact of different types of immobilization on daily life [17]. Recently, CARUSO et al. evaluated 74 adult patients using DASH, Mayo wrist, and Mayo elbow score at 1, 4, 12 weeks after adequate reduction of dorsally deviated DRF by comparing above-elbow and below-elbow cast. They concluded that patients treated by below-elbow cast have radiological and functional scores comparable to those treated with above-elbow cast with fewer complications secondary to immobilization of the elbow joint. Some points require attention in this study. First, both groups had similar DASH scores (4 weeks, BE: 71.7 vs. AE: 72), suggesting that both immobilizations were equally restrictive, which is very different from our findings (DASH 2 weeks; BE: 45.01 vs. AE:70.4; p < 0.01). Second, the main outcome chosen by the author was radiographic, limiting conclusions about the function of the upper limb between groups. Third, only two patients (one for each group) had loss of fracture reduction and were excluded from analysis. Despite including only dorsally deviated extraarticular fractures, the author found rates of loss of reduction much lower than ours (about 50%). Finally, their registration was performed almost two years after the start of the study [19].

Different constructions of immobilization, involving or not the elbow, have been recently evaluated by several authors [17–20]. In all, the primary outcome was the maintenance of radiographic parameters after adequate reduction and immobilization. Most included stable and unstable fractures. None found a significant difference in maintaining the reduction of DRF in adults. Despite the radiographic outcome being secondary, our results showed a similar reduction loss rate between the groups, corroborating the recent literature data. Patient characteristics, such as increasing age, is a good predictor of the loss of alignment during cast treatment. In agreement with recent literature, we found high levels of reduction loss rates in the population over 60 years old regardless of the treatment received [42].

Only a few studies used a VAS to assess wrist pain in nonsurgical treatment of DRF [7,45]. None showed a statistically significant difference in wrist pain, regardless of the time of assessment. Our findings are in agreement with the literature. Although the functional outcome with the PRWE questionnaire is desirable for DRF research, no comparable study with an AE cast was found. A recent study that compared 2 BE cast techniques for conservative treatment of DRF found that PRWE indices varied between 30–36 points (3 months), which was similar to this study [7]. A few articles measured handgrip strength after conservative treatment of wrist fractures. According to data, strength recovery varies from 40 to 100% compared to the contralateral side [7,9,13,46]. CHUNG & HAAS evaluated the handgrip strength of patients operated after DRF. According to the author it is necessary for the handgrip strength be at least 65% of the contralateral side in order to achieve patient satisfaction [47]. We obtained strength recovery above the minimum (AE: 69.6%; BE: 75.4%) at the 6-month evaluation.

One surprising finding was that 4-week elbow blocking did not impact the elbow’s range of motion and pain (long- and short-term evaluation), which differ from another recent study [19]. Shoulder pain is a common clinical finding in the non-surgical treatment of DRF. However, only recently has this been reported as a complication [17,48]. We found ipsilateral shoulder pain greater than 20 points (a clinically significant difference) in VAS for more than 3 consecutive visits in 17 (AE) vs. 9 (BE) participants. PARK et al. also found an increased incidence of shoulder pain between the long cast groups (64%) vs. short cast (28%) (p < 0.05), however without mentioning the form of measurement and pain intensity [17]. In our study, although all participants were instructed and encouraged to perform pendular exercises and active shoulder movement, we believe that patients treated with AE cast had more changes in the normal kinematics of the scapula that appear to place additional stress on the proximal segments, which may have contributed to the increased shoulder pain incidence [49]. Another factor that may have impacted the results was the difference in weight casts (BE: 350 g vs. AE: 600 g). In most cases the pain was reversed with medication and active and passive exercises. Some needed physical therapy follow-up. These results were previously defined as a secondary outcome, which may lead to an overestimation of the results.

It is established in the literature that objective outcomes, such as range of motion, grip strength or radiographic parameters, can lead to mistaken conclusions of the results, as they may not reliably reflect possible limitations in a patient’s daily activities. Accordingly, outcomes should be centered on the subject of the intervention. This establishes a paradigm shift from surgeon-centered care to patient-centered care [35,50,51]. We adopted a self-assessment questionnaire for the primary outcome, instead of objective assessments, as the results of the patients’ treatments. Consequently, the results of this study aim to improve the clinical condition and general quality of life for patients. Many authors have confirmed the validity and reliability of the DASH questionnaire in assessing function and disability in patients with deviated DRF [52–56]. Arora demonstrated the absence of a relation regarding functional performance and radiographical measures in the elderly [57]. A fact that is clear in this sample is that patients treated conservatively had good functional performance at the 24-week follow up, even when accepting some fracture displacement. In our opinion, there may be some space for non-operative treatment, especially in patients over 60 years with no higher levels of pre-injury activity and when cost-effectiveness is considered central in the decision-making process, as fees are considerable higher on more complex treatments, such as open reduction and internal fixation [58,59]. In addition, our results show that patients with AE cast have more complaints, especially due to shoulder pain. With this in mind, it may be reasonable to abandon elbow blocking on DRF treatment. When deciding about one treatment or the other, the concept of effectiveness (benefits and harms) is also an important consideration in a conservative treatment scenario.

Our study strengths are: 1) an *a priori* protocol [21] and registration; 2) the only RCT that uses DASH as the primary outcome throughout treatment; 3) a reasonable sample size; 4) low follow-up losses; 5) broad inclusion criteria (stable and unstable fractures); 6) inclusion of functional and surrogate outcomes (PRWE, ROM, radiographical measures, VAS); 7) blinded assessments; and 8) external, public funding (no COI from industry). Study limitations include: 1) consideration of both extra-articular and intra-articular fractures together; 2) the possible need for longer follow-up to assess articular degenerative disease; 3) non-everyday methods for fracture reductions were performed in the operating room; 4) our results may not be applicable for young, active adults; 5) this was a single-center study; and 6) change of the primary outcome during recruitment phase in relation to the published protocol and registration of the research.

## Conclusions

In conclusion, this study did not demonstrate a difference between above-elbow and below-elbow cast in terms of the self-reported DASH outcome at 6 months in non-surgical treatment of deviated distal radius fractures. However, below-elbow casting is less debilitating during the treatment period, provides comparable performance in maintaining the reduction, and has fewer minor adverse effects than above-elbow casting.

## Supporting information

S1 Checklist CONSORT checklist. (DOC)

S1 Appendix Preliminary analysis with the t-test to assess DASH outcome and additional analyses of the data. (DOCX)

S1 Data (XLSX)

S1 File SI trial protocol local committee. (DOCX)
