# Supplementary material for: No benefit for elbow blocking on conservative treatment of distal radius fractures: A 6-month randomized controlled trial
Source: PLoS One. 2021 Jun 10;16(6):e0252667. doi: 10.1371/journal.pone.0252667 (PMC8191961; doi:10.1371/journal.pone.0252667)
Supplement: S1 Appendix — (DOCX) [file pone.0252667.s002.docx]

**Preliminary analysis with the t-test to assess DASH outcome and additional analyses of the data**

We performed a preliminary analysis with the t-test to assess the primary outcome to justify the sample size.

**Table. The primary analysis of DASH.**

|  | **N** | **Mean** | **SD** | **95%CI** | **P-value** |
| --- | --- | --- | --- | --- | --- |
| **2W** |  |  |  |  | <0.001 |
| AE | 62 | 70.40 | 20.07 | 65.30 to 75.50 |  |
| BE | 63 | 45.26 | 24.70 | 39.04 to 51.48 |  |
| **6W** |  |  |  |  | 0.469 |
| AE | 64 | 37.12 | 19.73 | 32.20 to 42.05 |  |
| BE | 64 | 34.28 | 24.41 | 28.18 to 40.37 |  |
| **8W** |  |  |  |  | 0.974 |
| AE | 60 | 24.81 | 16.67 | 20.51 to 29.12 |  |
| BE | 63 | 24.70 | 21.23 | 19.35 to 30.05 |  |
| **12W** |  |  |  |  | 0.413 |
| AE | 57 | 15.09 | 12.79 | 11.7 to 18.49 |  |
| BE | 56 | 17.82 | 21.34 | 12.11 to 23.54 |  |
| **24W** |  |  |  |  | 0.851 |
| AE | 59 | 9.42 | 10.23 | 6.75 to 12.08 |  |
| BE | 58 | 9.82 | 12.88 | 6.44 to 13.21 |  |

Student’s t-test

95% CI – 95% Confidence interval for mean

We describe these analyses that were not stated in the published protocol but were reported in the article.

We used the Chi-Square test to measure the relationship between "Reduction Loss" with "Age".

**Table. Distribution of relative frequency of DRF according to age (< or > 60 years).**

|  | | AE | | BE | | Total | | P-value |
| --- | --- | --- | --- | --- | --- | --- | --- | --- |
|  |  | N | % | N | % | N | % |  |
| **Age** | **< 60 y** | 23 | 35.9% | 28 | 43.8% | 51 | 39.8% | 0.367 |
|  | **≥ 60 y** | 41 | 64.1% | 36 | 56.3% | 77 | 60.2% |  |

**Table. Relationship between "Reduction Loss" with "Age".**

| Age | | | No reduction loss | | Reduction loss | | Total | | RR | P-value |
| --- | --- | --- | --- | --- | --- | --- | --- | --- | --- | --- |
|  |  |  | N | % | N | % | N | % |  |  |
|  | **AE** | **< 60 y** | 18 | 62.1% | 5 | 14.3% | 23 | 35.9% | 0.30 | <0.001 |
|  |  | **≥ 60 y** | 11 | 37.9% | 30 | 85.7% | 41 | 64.1% | (0.16 to 0.54) |  |
|  | **BE** | **< 60 y** | 17 | 56.7% | 11 | 32.4% | 28 | 43.8% | 0.61 | 0.050 |
|  |  | **≥ 60 y** | 13 | 43.3% | 23 | 67.6% | 36 | 56.3% | (0.38 to 1.00) |  |
|  | **Both** | **< 60 y** | 35 | 59.3% | 16 | 23.2% | 51 | 39.8% | 0.46 | <0.001 |
|  |  | **≥ 60 y** | 24 | 40.7% | 53 | 76.8% | 77 | 60.2% | (0.31 to 0.66) |  |

We found a statistical relationship between "Reduction Loss" with "Age". We note that the reduction loss is greater in the age group above 60 years old. In the AE group, the index of people over 60 years old was 37.9% among people without loss of reduction against 85.7% among people with loss; RR= 0.3 (0.16 – 0.54); p-value <0.001. In the BE group, the indices were 43.3% and 67.6%, respectively; RR = 0.46 (0.31 – 0.66); p-value = 0.050.
