# Supplementary material for: No benefit for elbow blocking on conservative treatment of distal radius fractures: A 6-month randomized controlled trial
Source: PLoS One. 2021 Jun 10;16(6):e0252667. doi: 10.1371/journal.pone.0252667 (PMC8191961; doi:10.1371/journal.pone.0252667)
Supplement: S1 File — (DOCX) [file pone.0252667.s004.docx]

***
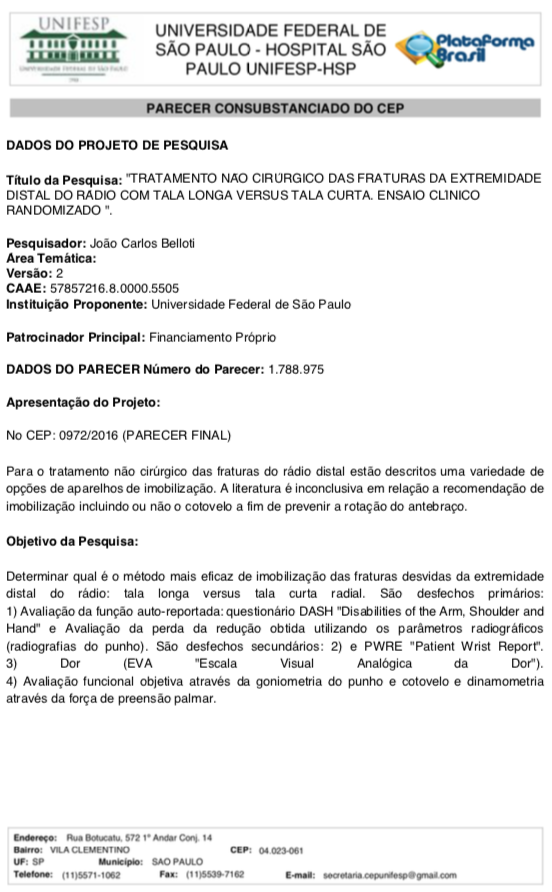
***

***
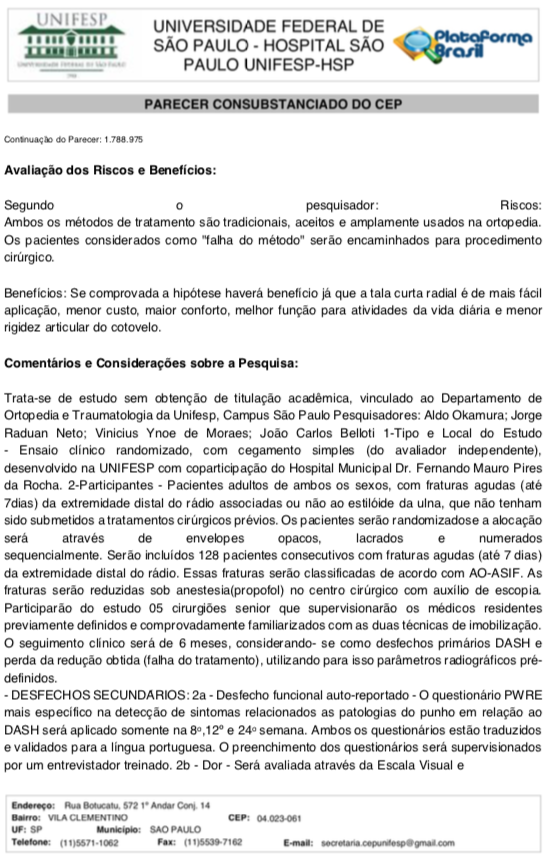
***

***
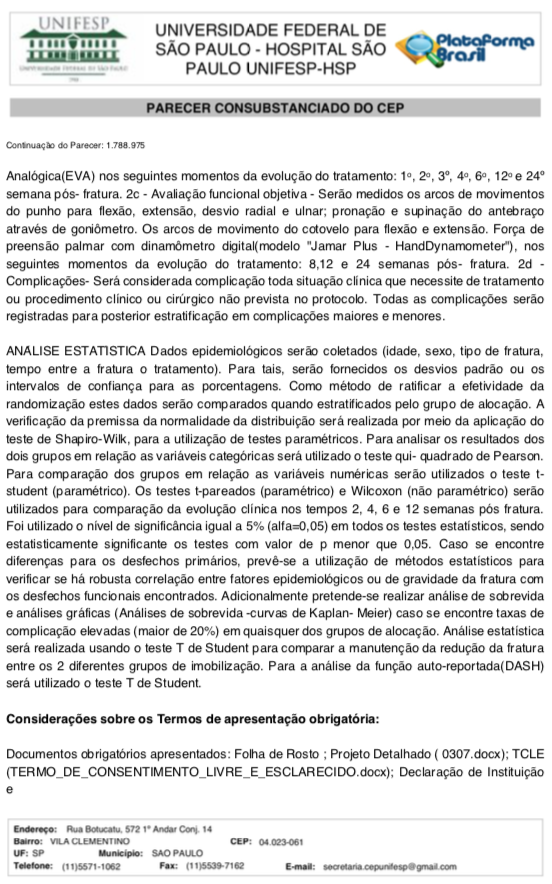
***

***
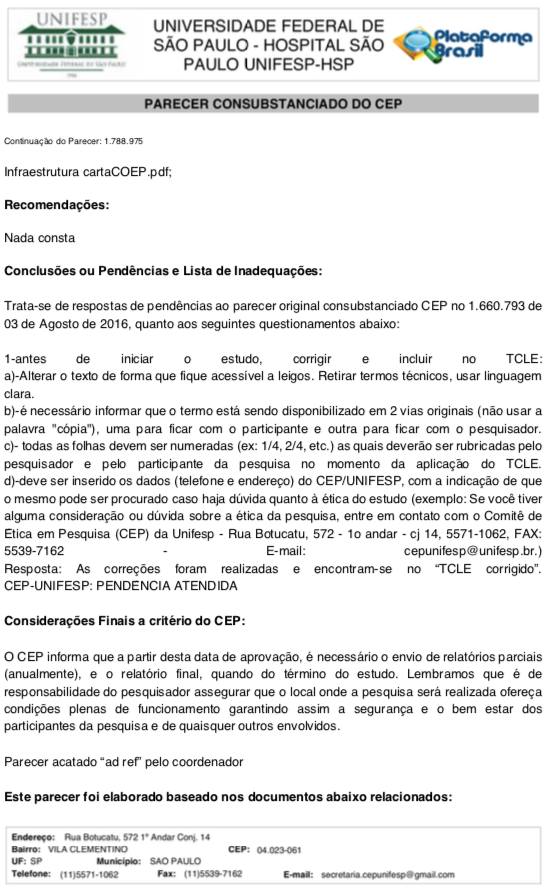
***

***
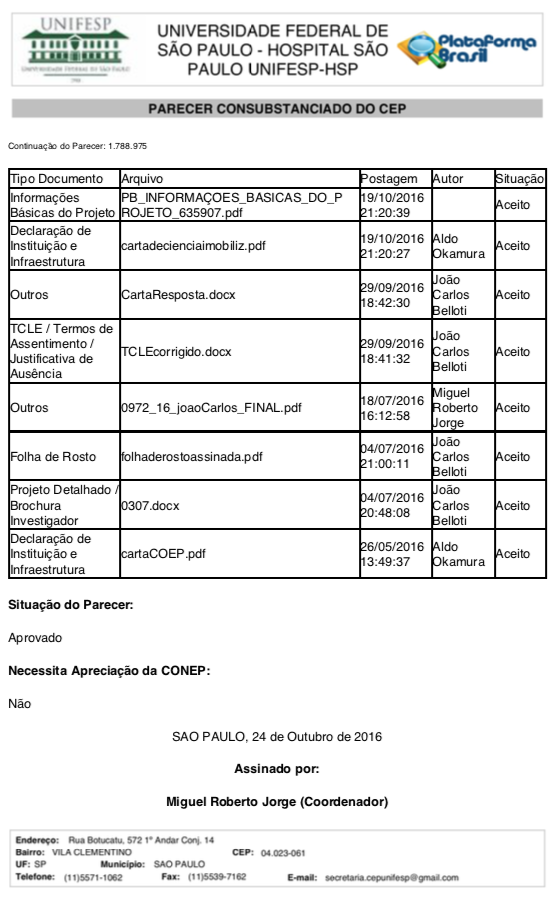
***

***English version approved by local ethics committee***

***Research Title:*** *Above-versus below-elbow casting for conservative treatment of distal radius fractures: a randomized controlled trial*

***Research Presentation:***

No CEP: 0972/2016 (FINAL DECISION)

*A variety of immobilization device options are described for non-surgical treatment of distal radius fractures. The literature is inconclusive regarding the recommendation for immobilization whether or not the elbow is included in order to prevent forearm rotation.*

***Objectives***

*This study aimed to evaluate the best method of immobilization between above-elbow (AE) and below-elbow (BE) cast groups. Primary outcomes are:1) Evaluation of the self-reported function: DASH questionnaire "Disabilities of the Arm, Shoulder and Hand" and Evaluation of the loss of reduction obtained using radiographic parameters (wrist radiographs). Secondary outcomes are: 2) and PWRE "Patient Wrist Report". 3) Pain (EVA "Visual Analog Pain Scale"). 4) Objective functional assessment using wrist and elbow goniometry and dynamometry using handgrip strength.*

***Risks and Benefits***

*Both treatment methods are traditional, accepted and widely used in orthopedics. Patients considered "method failure" will be referred for surgical procedure.*

*Benefits: If the hypothesis is proven, there will be a benefit since the short radial splint is easier to apply, less costly, more comfortable, better function for activities of daily living and less stiff elbow joint.*

***Research Comments and Considerations:***

*Department of Orthopedics and Traumatology at UNIFESP, Campus São Paulo Researchers: Aldo Okamura; Jorge Raduan Neto; Vinicius Ynoe de Moraes; João Carlos Belloti*

*1-Study Type and Location- Randomized clinical trial, with simple blinding (independent evaluator), developed at UNIFESP with co-participation of the Municipal Hospital Dr. Fernando Mauro Pires da Rocha.*

*2-Participants - Adult patients of both sexes, with acute fractures (up to 7 days) of the distal end of the radius associated or not with the ulna styloid, who have not undergone previous surgical treatments. Patients will be randomized and the allocation will be through opaque, sealed and numbered envelopes sequentially. 128 consecutive patients with acute fractures (up to 7 days) of the distal end of the radius will be included. These fractures will be classified according to AO-ASIF. Fractures will be reduced under anesthesia (propofol) in the operating room under radioscopy. Five senior surgeons will participate in the study who will supervise previously defined resident physicians and have proven to be familiar with both immobilization techniques. The clinical follow-up will be 6 months, considering primary DASH outcomes and loss of the reduction obtained (treatment failure), using pre-defined radiographic parameters.*

*- SECONDARY OUTCOMES: 2a - Self-reported functional outcome - PWRE questionnaire will be applied in the 8th, 12th and 24th week. 2b - Pain - Will be assessed by Visual Scale and Analog (VAS) in the following moments of the treatment evolution: 1st, 2nd, 3rd, 4th, 6th, 12th and 24th week after fracture. 2c - Objective functional evaluation - Wrist movement arcs for flexion, extension, radial and ulnar deviation will be measured; pronation and supination of the forearm through a goniometer. The arches of movement of the elbow for flexion and extension. Strength of handgrip with digital dynamometer ("Jamar Plus – Hand Dynamometer" model), in the following moments of the treatment evolution: 8,12 and 24 weeks post-fracture. 2d - Complications- Any clinical situation that requires treatment or clinical or surgical procedure not provided for in the protocol will be considered a complication. All complications will be recorded for further stratification into major and minor complications.*

***STATISTICAL ANALYSIS***

*Epidemiological data will be collected (age, sex, type of fracture, time between fracture and treatment). For such, standard deviations or confidence intervals for percentages will be provided. As a method of confirming the effectiveness of randomization, these data will be compared when stratified by the allocation group. The verification of the premise of the normality of the distribution will be carried out through the application of the Shapiro-Wilk test, for the use of parametric tests. Pearson's chi-square test will be used to analyze the results of the two groups in relation to the categorical variables. To compare the groups in relation to the numerical variables, the t-student test (parametric) will be used. The paired t-tests (parametric) and Wilcoxon (non-parametric) will be used to compare the clinical evolution at 2, 4, 6 and 12 weeks after fracture. The significance level of 5% (alpha = 0.05) was used in all statistical tests, with tests with a p-value less than 0.05 being statistically significant. If differences are found for primary outcomes, the use of statistical methods is expected to verify whether there is a robust correlation between epidemiological factors or fracture severity with the functional outcomes found. Additionally, it is intended to perform survival analysis and graphical analysis (Analysis of survival - Kaplan-Meier curves) if high complication rates (greater than 20%) are found in any of the allocation groups. Statistical analysis will be performed using Student's T test to compare the maintenance of fracture reduction between the 2 different immobilization groups. Student's t test will be used for the analysis of the self-reported function (DASH).*


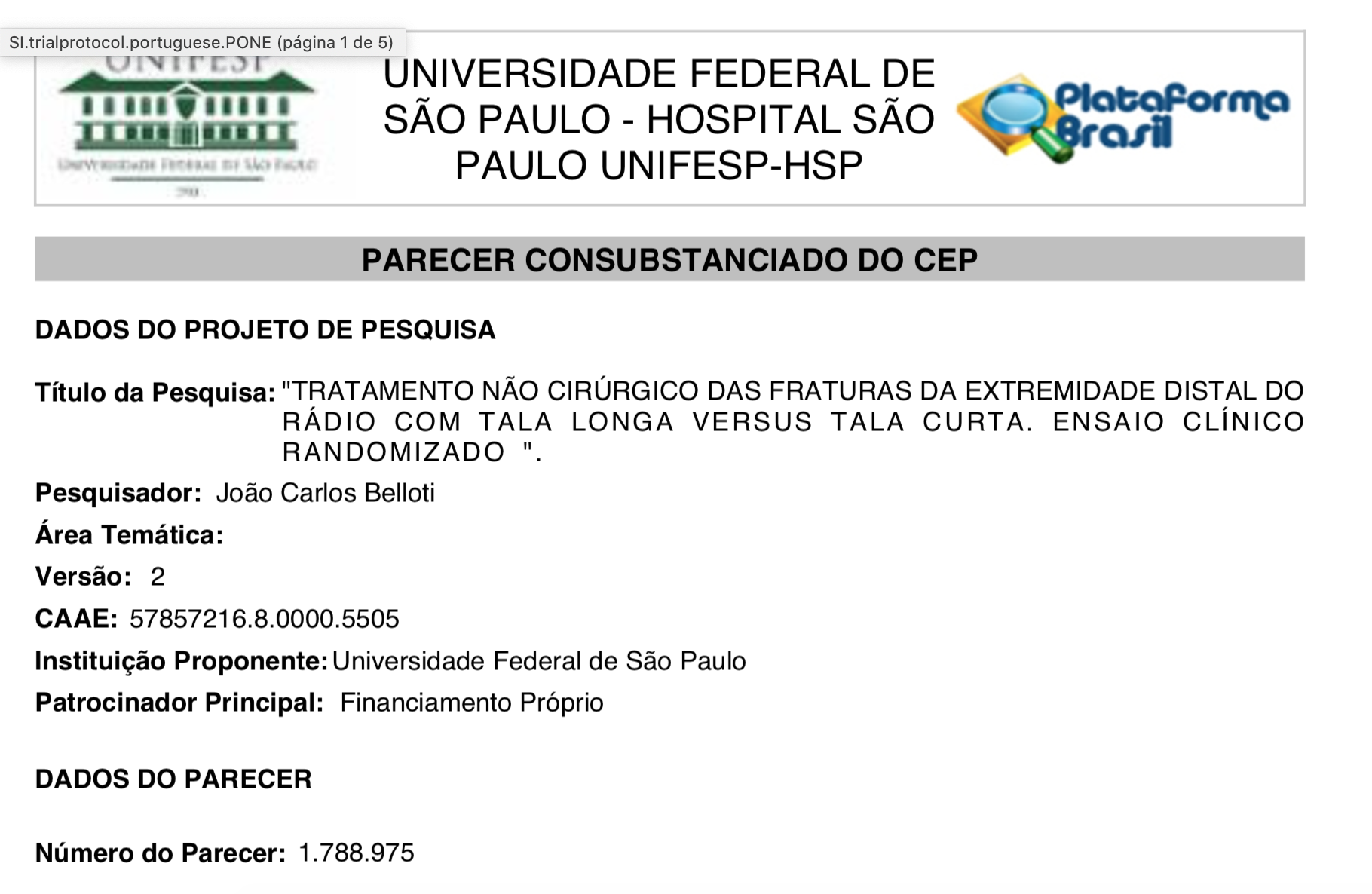


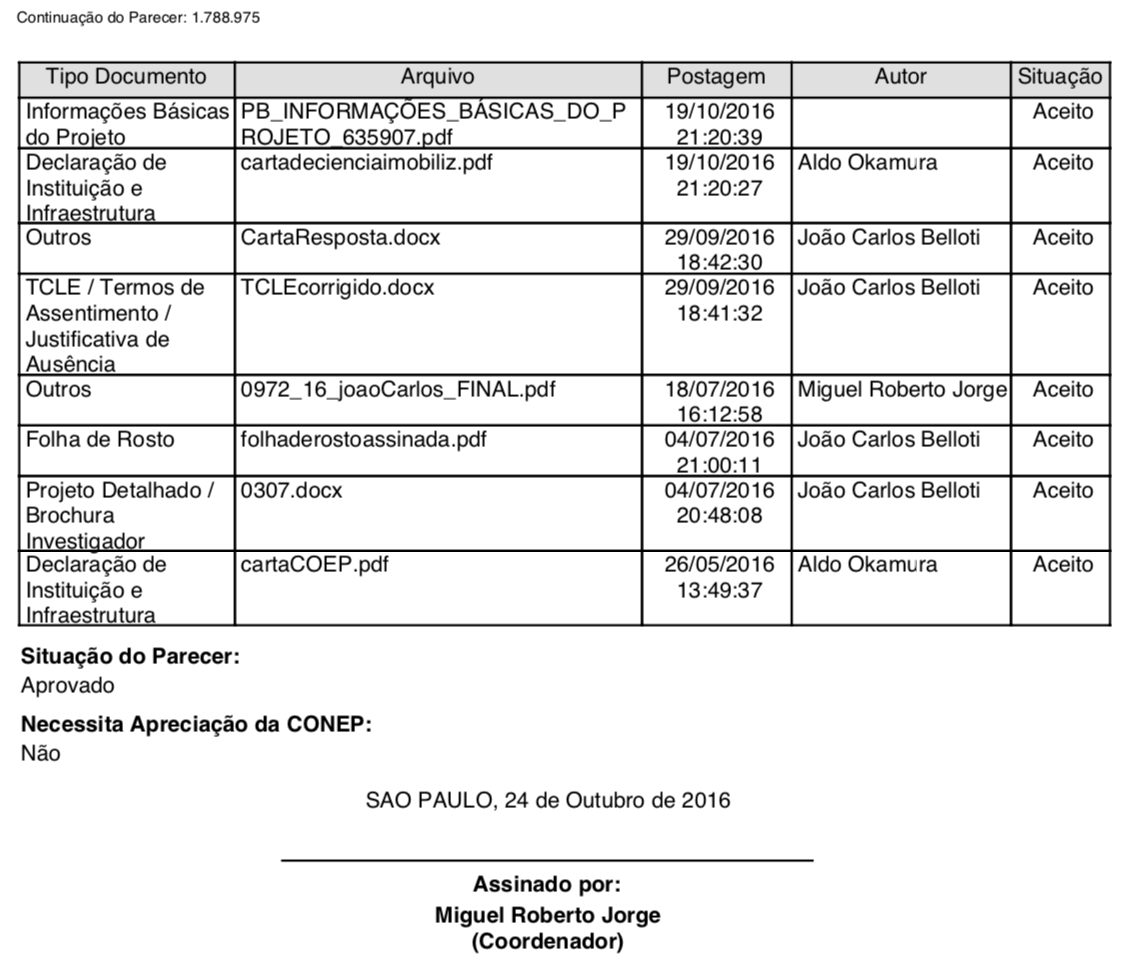


Detailed Project

(page10-portuguese.

Page 21-english)

accepted

**Status: Approved**

PROJETO DETALHADO (04/07/2016)

RESUMO

Introdução- Para o tratamento não cirúrgico das fraturas do rádio distal estão descritos uma variedade de opções de aparelhos de imobilização. A literatura é inconclusiva em relação a recomendação de imobilização incluindo ou não o cotovelo a fim de prevenir a rotação do antebraço para as fraturas do rádio distal. O objetivo deste estudo é avaliar o melhor método de imobilização entre os grupos tala longa vs. tala curta ao final do seguimento de seis meses.

Método– Trata-se de um Ensaio Clínico Randomizado, envolvendo duas intervenções não cirúrgicas. Os pacientes serão alocados de forma randomizada utilizando-se envelopes opacos e lacrados. Serão incluídos 128 pacientes consecutivos com fraturas agudas (até 7 dias) da extremidade distal do rádio. O seguimento clínico mínimo será de 6 meses, considerando-se como desfechos primários avaliação de parâmetros a função medida pelo *Disabilities of the Arm, Shoulder and Hand*-DASH e radiográficos (radiografias do punho). São desfechos secundários: dor medida pela Escala Visual Analógica, avaliação funcional objetiva (goniometria e dinamometria), questionário PRWE, taxa de complicações e de falha do método, respeitando o princípio de intenção de tratar. Cálculo Amostral - Para detectar uma diferença de 10% no DASH e antecipando uma perda de 10% no seguimento serão incluídos 128 pacientes.

ENUNCIADO DO PROBLEMA

Embora as fraturas da extremidade distal do rádio estejam entre as mais frequentes do membro superior^1^, o melhor método de tratamento, classificação e desfecho destas fraturas ainda não está totalmente definido^2,3,4,5^. Em relação ao tratamento conservador os estudos são controversos em relação a tipo de imobilização a ser aplicada após a redução inicial da fratura. A imobilizacão abaixo do cotovelo é de mais facil aplicação, menor custo, maior conforto, melhor função para atividades da vida diária e menor rigidez articular do cotovelo^6,7,8^. Já as imobilizações que incluem o cotovelo, prevenindo a rotação do antebraço, há maior estabilidade da fratura e menor risco de perda de redução e necessidade de remanipulação^9,10,11,12^. Outro estudo encontrou performance semelhante entre os métodos de imobilização na manutenção da redução inicial da fratura^13,14^.

Este estudo é baseado na hipótese de que a utilização de imobilização com tala radial curta em pacientes com fraturas da extremidade distal do rádio apresentarão melhores resultados para os desfechos funcionais auto-reportados, parâmetros radiográficos, e taxa de complicações quando comparados aos métodos de imobilização longa, ao final do acompanhamento de 6 meses.

Objetivo

Determinar qual é o melhor método de imobilização nos pacientes com fraturas da extremidade distal do rádio: tala longa *versus* tala curta radial. São desfechos primários: avaliação da função auto-reportada: questionário DASH –“*Disabilities of the Arm, Shoulder and Hand*”^15^ e avaliação de parâmetros radiográficos (radiografias do punho). São desfechos secundários: dor (EVA – “Escala Visual Analógica da Dor”)^16^, questionário de auto-avaliação PRWE^17^, avaliação funcional objetiva (goniometria e dinamometria), taxa de complicações e de falhas (*intention-to-treat principle*).

MATERIAL E MÉTODO

1-Tipo e Local do Estudo - Ensaio clínico randomizado, com cegamento simples (do avaliador independente), desenvolvido na UNIFESP com coparticipação do Hospital Municipal Dr. Fernando Mauro Pires da Rocha.

2-Participantes - Pacientes adultos de ambos os sexos, com fraturas agudas (até 7dias) da extremidade distal do rádio associadas ou não ao estilóide da ulna, que não tenham sido submetidos a tratamentos cirúrgicos prévios.

3-Critérios de inclusão: Todos os pacientes com fratura aguda desviada do rádio distal passíveis de redução fechada.

Serão consideradas fraturas desviadas as fraturas que apresentarem, antes da manipulação, a perda de pelo menos 1 dos critérios abaixo. O lado contralateral será utilizado como referência.

- Altura radial - aceito perda de até 3 mm

- Inclinação radial - aceito perda de até 5°

- Inclinação volar - aceito até 10° dorsal

- Variância ulnar – aceito diferença de até 3 mm

- Fragmento articular com desvio – aceito até 2 mm

Serão consideradas fraturas redutíveis aquelas que após a redução e imobilização mantiverem os parâmetros acima descritos.

Todos os pacientes incluídos no estudo serão classificados de acordo com a classificação AO- ASIF^18,19^.

Classificação “AO-ASIF”^18,19^:

A - Fratura extra-articular:

A1- Só a ulna

A2 - Terço distal do rádio, sem cominuição nem impactação

A3 **-** Terço distal do rádio, com cominuição ou impactação

B - Fratura intra-articular marginal (se mantém o bloco epimetafisário):

B1-Estilóides

B2**-**Fragmento dorsal (Barton)

B3 - Fragmento volar (Barton invertida)

C-Fratura intra-articular cominutiva:

C1 - Se mantém a congruêncla articular, sem cominuição metafisária

C2 **-** Perda da congruência articular, sem cominuição metafisária

C3 - Cominuiçãao metafisária no rádio e ulna e fratura articular com perda da congruência

4-Critérios de exclusão – Serão excluídos do estudo os pacientes que apresentarem 1 ou mais dos seguintes critérios:

- Pacientes com fraturas marginais ou com mecanismo de cisalhamento (B2 e B3 AO-ASIF).

- Pacientes com fraturas irredutíveis.

- Fraturas ipsilaterais do membro superior.

- Politraumatizados.

- História prévia de doença degenerativa ou traumática na articulação do punho acometido ou contralateral: reconhecidas pela história clinica ou diagnosticadas por radiografias (frente e perfil do punho).

- Fratura bilateral, fraturas expostas ou associadas às lesões tendíneas ou neurovasculares.

- Pacientes com doenças sistêmicas ou lesões traumáticas associadas à fratura que restrinjam a aplicação dos métodos ou a avaliação dos resultados.

- Déficit cognitivo que não permita ao paciente compreender os componentes da avaliação funcional.

- Recusa ao Termo Consentimento.

INTERVENÇÃO:

1- Atendimento inicial - Os pacientes atendidos nos pronto-socorros serão submetidos ao exame clínico e radiográfico, com radiografias bilaterais dos punhos nas posições posteroanterior (PA) e perfil (P). Após aplicação dos critérios de inclusão e exclusão os indivíduos elegíveis serão informados sobre a natureza e objetivo do estudo, mediante a leitura do “Termo de Consentimento Livre e Esclarecido” e após assinatura do mesmo serão cadastrados. Os pacientes com fraturas desviadas serão encaminhados para um dos pesquisadores responsáveis que fará o agendamento no centro cirúrgico para anestesia (sedação) e redução fechada da fratura sob controle fluoroscópico. Os pacientes que tiverem fraturas passíveis de redução fechada serão randomizados através de envelope selado para um dos dois métodos do estudo. Quando a fratura for irredutível o paciente será excluído do estudo. Se a redução da fratura for considerada inadequada uma segunda redução será feita utilizando a mesma construção de tala.

2- Anestesia – Os pacientes serão submetidos a sedação intravenosa no centro cirúrgico.

3-Método de Imobilização- Participarão do estudo 05 cirurgiões de mão *senior* que supervisionarão os médicos residentes previamente definidos e comprovadamente familiarizados com as duas técnicas de imobilização. O material necessário para a aplicação das duas técnicas estará disponível na sala cirúrgica. Após a sedação o paciente será submetido à redução fechada da fratura através da técnica de tração e contra-tração. Inicialmente, todos os pacientes receberão a tala curta radial que será realizada com gesso de largura 20cm cortado para encaixe do polegar. A tala será aplicada no aspecto radial do punho cobrindo a porção volar e dorsal do radio até o cotovelo. Os pacientes randomizados para tala longa receberão uma complementação da imobilização com uma tala de largura 15cm no aspecto ulnar do antebraço que se inicia no 1/3 médio do antebraço e vai até a axila. O cotovelo será imobilizado em 90 graus, posição neutra para bloqueio da prono-supinação. Faixas de algodão e malha tubular serão usadas em ambas as imobilizações. Independentemente da imobilização adotada todos os punhos serão posicionados em leve flexão e desvio ulnar. Os pacientes serão estimulados a movimentar ativamente os dedos e o ombro ipsilateral.

5- Acompanhamento:

Ambos os grupos de tratamento serão imobilizados por 6 semanas e orientados a retornar no ambulatório na semana seguinte a redução da fratura e nas 2^o^,4^o^ e 6^o^ semana pós fratura. Os pacientes com imobilização longa permanecerão 4 semanas com a tala axilopamar, seguida de 2 semanas de imobilização radial curta. Após retirada da imobilização (6^o^ semana) os pacientes retornarão com 8, 12 e 24 semanas pós fratura.

DESFECHOS CLÍNICOS

A avaliação funcional auto-reportada(DASH), radiográfica, e avaliação funcional objetiva serão realizadas por profissionais não ligados diretamente ao estudo. Para os desfechos com 8,12 e 24 semanas os avaliadores serão cegos aos grupos de alocação dos pacientes. O seguimento clínico mínimo será de 24 semanas, considerando-se para avaliação dos resultados os seguintes parâmetros:

1- DESFECHOS PRIMÁRIOS:

1a - Desfechos funcionais auto-reportados – O status funcional será avaliado através do questionário DASH nos seguintes momentos: 2^o^, 6^o^, 8º, 12º e 24^o^ semanas pós- fratura. Todos os questionários serão supervisionados por um entrevistador treinado. O questionário foi elaborado pela Academia Americana e consiste em 37 perguntas relacionadas a função da mão, punho, cotovelo e ombro. Está validada para a língua portuguesa^20^.

1b – Parâmetros radiográficos – Mensuração dos desvios angulares ^21,22,23^ e lesões associadas. Serão aferidos a altura radial, inclinação radial, inclinação volar, variância ulnar e fragmento articular, considerados para a avaliação as radiografias nas posições posteroanterior e perfil, nos seguintes momentos da evolução do tratamento: inicial, pós-redução imediata(nas fraturas desviadas), 1^o^, 2^o^, 3º, 4^o^, 6^o^, 8º, 12º e 24º semana da fratura. As mensurações serão realizadas por dois pesquisadores de forma independente.

2- DESFECHOS SECUNDÁRIOS:

2a - Avaliação funcional objetiva - Serão medidos os arcos de movimentos do punho para flexão, extensão, desvio radial e ulnar; pronação e supinação do antebraço; flexão e extensão do cotovelo através de goniômetro. Força de preensão palmar com dinamômetro digital (modelo “Jamar Plus - Hand Dynamometer”), nos seguintes momentos da evolução do tratamento: 8,12 e 24 semanas pós- fratura.

2b - Dor – A dor no punho, ombro e cotovelo serão avaliadas através da Escala Visual e Analógica (EVA) nos seguintes momentos da evolução do tratamento: 1^o^, 2^o^, 3º, 4^o^, 6^o^, 8º, 12º e 24º semana pós- fratura.

2c – questionário funcional PRWE – Será avaliado na 8º, 12º e 24º semana pós fratura.

2c - Complicações- Será considerada complicação toda situação clínica que necessite de tratamento ou procedimento clínico ou cirúrgico não prevista no protocolo. Todas as complicações serão registradas para posterior estratificação em complicações maiores e menores.

2d - Falha do método – Será considerado falha do método toda complicação que implique na interrupção ou mudança do método de tratamento com o método previamente randomizado.

ANÁLISE ESTATÍSTICA – ANÁLISE POR INTENÇÃO DE TRATAR

Dados epidemiológicos serão coletados (idade, sexo, tipo de fratura, tempo entre a fratura o tratamento). Para tais, serão fornecidos os desvios padrão ou os intervalos de confiança para as porcentagens. Como método de ratificar a efetividade da randomização estes dados serão comparados quando estratificados pelo grupo de alocação.

A verificação da premissa da normalidade da distribuição será realizada por meio da aplicação do teste de Shapiro-Wilk, para a utilização de testes paramétricos. Para analisar os resultados dos dois grupos em relação as variáveis categóricas será utilizado o teste qui-quadrado de Pearson. Para comparação dos grupos em relação as variáveis numéricas serão utilizados o teste t-student (paramétrico). Os testes t-pareados (paramétrico) e Wilcoxon (não paramétrico) serão utilizados para comparação da evolução clínica nos tempos 2, 3, 4, e 6 semanas pós fratura e posteriormente com 8, 12 e 24 semanas de pós fratura. Foi utilizado o nível de significância igual a 5% (alfa=0,05) em todos os testes estatísticos, sendo estatisticamente significante os testes com valor de P menor que 0,05.

Caso se encontre diferenças para os desfechos primários, prevê-se a utilização de métodos estatísticos para verificar se há robusta correlação entre fatores epidemiológicos ou de gravidade da fratura com os desfechos funcionais encontrados. Adicionalmente pretende-se realizar análise de sobrevida e análises gráficas (Análises de sobrevida – curvas de Kaplan-Meier) caso se encontre taxas de complicação elevadas (maior de 20%) em quaisquer dos grupos de alocação.

Análise estatística será realizada usando o teste T de Student para comparar a manutenção da redução da fratura entre os 2 diferentes grupos de imobilização.

Análise comparando a porcentagem de perda de redução entre os dois grupos em relação as classificações AO-ASIF.

Para a análise da função auto-reportada(DASH) será utilizado o teste T de Student.

Os pacientes que, por qualquer motivo, tiverem falha no tratamento ou necessitaram de intervenções adicionais, serão acompanhados e seus resultados computados no seu grupo primário de alocação (princípio de intenção de tratar). É previsto cegamento dos dados para análise estatística, por um estatístico que não conhece os objetivos e desfechos de interesse.

RANDOMIZAÇÃO E MASCARAMENTO

A decisão para inclusão de pacientes nos grupos de alocação seguirão o seguinte método de randomização: envelopes serão numerados em sua face externa com números consecutivos, sendo que o sorteio do método para cada envelope será feito ao acaso e de forma consecutiva por meio de software de randomização (http://www.randomizer.org/). O envelope somente será aberto na sala cirúrgica após a verificação do critério de redutibilidade da fratura. O procedimento de randomização estará a cargo de pessoa não diretamente ligada ao estudo.

CÁLCULO AMOSTRAL

Com base em uma análise inicial calculamos que para ser capaz de detectar uma diferença de 10% na pontuação do questionário de deficiência do braço, ombro e mão (DASH) e antecipando uma perda de pacientes de 10% no acompanhamento seria necessário um total de 128 fraturas do rádio distal.

ABREVIAÇÕES

AO: Arbeitsgemeinschaft für Osteosynthesefragen Foundation; ASIF: Association for the Study of Internal Fixation; CEP: Comitê de Ética em Pesquisa; DASH: Disabilities of Arm, Shoulder and Hand; EPM: Escola Paulista de Medicina; EVA: Escala Visual Analógica da Dor; P: Perfil; PA: Posteroanterior; UNIFESP: Universidade Federal de São Paulo.

DETALHES DOS AUTORES

1. Disciplina de Cirurgia da Mão e Membro Superior do Departamento de Ortopedia e Traumatologia da Universidade Federal de São Paulo- UNIFESP/EPM

CONTRIBUIÇÃO DOS AUTORES

Aldo Okamura, Jorge Raduan Neto, Vinicius Ynoe de Moraes, Flavio Faloppa e João Carlos Belloti desenvolveram o protocolo do estudo e são responsáveis pelo recrutamento, realização do tratamento cirúrgico e avaliação dos paciente incluídos neste estudo.

CONFLITOS DE INTERESSE

Os autores declaram que não existe conflito de interesse.

REFERÊNCIAS

1-Fernandez DL, Palmer AK. Fractures of the distal radius. In : The Green DP, Hotchkiss RN, Pederson WC, editor(s). Greens Operative Hand Surgery. 4th Edition. New York: Churchill Livingstone: 929-985,1999.

2- Cui Z, Pan J, Yu B, Zhang K, Xiong X. Internal versus external fixation for unstable distal radius fractures: an up-to-date meta-analysis. Int Orthop. 2011 Sep;35(9):1333-41.

3- Diaz-Garcia RJ, Oda T, Shauver MJ, Chung KC. A systematic review of outcomes and complications of treating unstable distal radius fractures in the elderly. J Hand Surg Am. 2011 May;36(5):824-35 e2.

4- Jupiter JB, Fernandez DL. Comparative classification for fractures of the distal end of the radius. J Hand Surg 1997; 22A:563–571.

5- Lichtman DM, Bindra RR, Boyer MI, Putnam MD, Ring D, Slutsky DJ et al. Treatment of distal radius fracture. J Am Acad Orthop Surg 2010;18:180-9.

6- Pool C. Colles’s fracture. A prospective study of treatment. J Bone Joint Surg 1973;55B:540–544.

7- Stewart HD, Innes AR, Burke FD. Functional cast-bracing for Colles’ fractures A comparison between cast-bracing and conventional plaster casts. J Bone Joint Surg 1984;66B:749 – 753.

8- Webb GR, Galpin RD, Armstrongs DG. Comparison of short and long arm plaster casts for displaced fractures in the distal third of the forearm in children. J Bone Joint Surg 2006; 88 A:9-17.

9- Hughston JC. Fractures of the forearm in children. J Bone Joint Surg Am. 1962;44:1678-930

10-Wahlstrom O. Treatment of Colles’ fracture A prospective comparison of three different positions of immobilization. Acta Orthop Scand 1982;53:225–228.

11- Sarmiento A, Pratt GW, Berry NC, Sinclair WF. Colles’ fractures Functional bracing in supination. J Bone Joint Surg 1975;57A:311–317.

12- Bunger C, Solund K, Rasmussen P. Early results after Colles’ fracture: functional bracing in supination vs dor- sal plaster immobilization. Arch Orthop Trauma Surg 1984;103:251–256.

13 - Bong MR, Egol KA, Leibman M, Koval KJ: A comparison of immediate postreduction splinting constructs for controlling initial displacement of fractures of the distal radius: A prospective randomized study of long- arm versus short-arm splinting. *J Hand Surg Am* 2006;31:766-770.

14- Bohm ER, Bubbar V, Hing KY, Dzus A. Above and below-the-elbow plaster cast for distal forearm fractures in children. J Bone J Surg Am. 2006; 88 A:1-8.

15- Hudak PL, Amadio PC, Bombardier C. Development of an upper extremity outcome measure: the DASH (disabilities of the arm, shoulder and hand) [corrected]. The Upper Extremity Collaborative Group (UECG). Am J Ind Med. 1996 Jun;29(6):602-8.

16- Revill SI, Robinson JO, Rosen M, Hogg MIJ. The reliability of a linear analogue for evaluating pain. Anaesthesia. 1976;31(9):1191-8.

17- Rodrigues EKS, Fonseca MCR, MacDermid JC. Brazilian version of yhe patient rated wrist evaluation (PRWE-Br): cross- cultural adaptation, internal consistency, test- retest reliability, and construct validity. J Hand Ther. 2015;28(1):69–76.

18- Müller ME (1996) CCF—Comprehensive Classification of Fractures I & II. M.E.Müller Foundation. Bern: MAO/ASIF Documentation Center.

19- Kreder HJ, Hanel DP, McKee M, Jupiter J, McGilivary G, Swiontowski MF: Consistency of AO fracture classification for the distal radius. J.Bone Joint Surg 78B:726-31,1996.

20 - Orfale AG, Araújo PM, Ferraz MB, Natour J. Translation into Brazilian Portuguese, cultural adaptation and evaluation of the reliability of the disabilities of the arm, shoulder and hand questionnaire. Braz J Med Biol Res. 2005;38:293-302. <https://doi.org/10.1590/S0100-879X2005000200018>

21- Kreder HJ, Hanel DP, McKee M, Jupiter J, McGilivary G, Swiontowski MF: X-ray film measurements for healed distal radius fractures. J Hand Surg [Am] 1996 May;21(3):532.

22- Sharpe F, Stevanovic M. Extra-articular distal radial fracture malunion. Hand Clin. 2005 Aug;21(3):469-87

23- Medoff RJ. Essential radiographic evaluation for distal radius fractures. Hand Clin 2005; 21:279-288.

**DETAILED PROJECT (JULY 04th, 2016)- English version**

ABSTRACT

BACKGROUND – A variety of cast options are available for the non-surgical treatment of distal radius fractures (DRF) in adults. However, the literature is inconclusive regarding the need to immobilize the elbow joint after reduction in order to prevent rotation of the forearm in order to maintain the reduction of DRF. This study aimed to evaluate the best method of immobilization between above-elbow (AE) and below-elbow (BE) cast groups at the end of six-month follow-up.

METHODS – This is a randomized clinical trial with parallel groups and a blinded evaluator. There are two non-surgical interventions: AE and BE. Patients will be randomly assigned. A hundred twenty-eight consecutive adult patients with acute (up to 7 days) displaced DRF. The primary outcome will be the Disabilities of the Arm, Shoulder and Hand Questionnaire (DASH) and the maintenance of reduction by evaluation of radiographic parameters. Secondary outcomes include function measured by Patient Rated Wrist Evaluation (PRWE), pain measured by the Visual Analogue Scale (VAS), objective functional evaluation (goniometry and dynamometry) and rate of complications. Evaluations will be performed at 1, 2, 3, 4, 6, 8, 12 and 24 weeks. For the Student´s t-test, a difference of 10 points in DASH score and an extra 10% for balancing follow up losses results in 128 patients.

**PROBLEM STATEMENT**

Although distal radius fractures (DRF) are among the most frequent fractures of the upper limb^1^, the best treatment method, the classification and outcome of these fractures is not yet fully defined^2,3,4,5^. Regarding the conservative treatment of the studies, they are controversial regarding the type of immobilization to be applied after the initial fracture reduction. Immobilization below-elbow is easier to apply, less costly, more comfortable, better function for activities of daily living and less elbow joint stiffness^6,7,8^. Immobilizations that include the elbow prevent forearm rotation, have greater fracture stability and less risk of loss and need for remanipulation^9,10,11,12^. Another study found similar performance among immobilization methods in maintaining the initial fracture reduction^13,14^.

This study is based on the hypothesis that below-elbow cast (BE) in patients with distal radius fractures provides better results for self-reported outcomes, radiographic parameters, and complication rate when compared to above-elbow cast (AE), at the end of the 6-month follow-up.

**OBJECTIVE**

Determine which is the best immobilization method in patients with DRF: AE vs. BE. The primary outcomes are: self-reported function assessment: DASH questionnaire - “Arm, Shoulder and Hand Deficiencies” ^15^ and assessment of radiographic parameters (wrist radiographs). Secondary outcomes are: pain (VAS - “Visual Analog Pain Scale”) ^16^, self-assessment questionnaire PRWE^17^, objective functional assessment (goniometry and dynamometry), rate of complications and failures (principle of intention to treat).

**METHODS**

1-Type and Location - Randomized clinical trial, with simple blinding (of the independent evaluator), developed at UNIFESP with co-participation of the Municipal Hospital Dr. Fernando Mauro Pires da Rocha.

2-Participants - Adult of both sexes, with acute fractures (up to 7 days) of the distal end of the radius associated or not with the ulna styloid, who have not undergone previous surgical treatments.

3-Inclusion criteria: All patients with acute fracture deviated from the distal radius subject to closed reduction.

Deviated fractures will be considered fractures that present, before manipulation, the loss of at least 1 of the criteria below. The contralateral side will be used as a reference.

- Radial height - loss of up to 3 mm is accepted

- Radial inclination - loss of up to 5 ° accepted

- Volar tilt - accepted up to 10 ° dorsal

- Ulnar variance - difference of up to 3 mm accepted

- Joint fragment with deviation - accepted up to 2 mm

Reducible fractures will be considered as those that, after reduction and immobilization, maintain the parameters described above.

All patients included in the study will be classified according to the AO-ASIF classification^18,19^.

“AO-ASIF” classification ^18,19^:

A - Extra-articular fracture:

A1- Only the ulna

A2 - Distal third of the radius, without comminution or impaction

A3 - Distal third of the radius, with comminution or impaction

B - marginal intra-articular fracture (the epimetaphyseal block is maintained):

B1-Stiloids

B2-Dorsal fragment (Barton)

B3 - Volar fragment (inverted Barton)

C-Comminuted intra-articular fracture:

C1 - The joint congruence is maintained, without metaphysical comminution

C2 - Loss of joint congruence, without metaphyseal comminution

C3 - Metaphyseal comminution in the radius and ulna and joint fracture with loss of congruence

4-Exclusion criteria - Patients with 1 or more of the following criteria will be excluded from the study:

- Patients with marginal fractures or with a shear mechanism (B2 and B3 AO-ASIF).

- Patients with irreducible fractures.

- ipsilateral fractures of the upper limb.

- Polytraumatized.

- Previous history of degenerative or traumatic disease in the affected or contralateral wrist joint: recognized by clinical history or diagnosed by radiographs (front and profile of the wrist).

- Bilateral fracture, fractures exposed or associated with tendon or neurovascular injuries.

- Patients with systemic diseases or traumatic injuries associated with the fracture that restrict the application of the methods or the evaluation of the results.

- Cognitive deficit that does not allow the patient to understand the components of the functional assessment.

- Consent Refusal*.*

**INTERVENTION**

1- Initial care - Patients seen in the emergency rooms will be submitted to clinical and radiographic examination, with bilateral radiographs of the wrists in the posteroanterior (PA) and lateral (P) positions. After applying the inclusion and exclusion criteria, eligible individuals will be informed about the nature and purpose of the study, by reading the “Informed Consent Form” and after signing it will be registered. Patients with deviated fractures will be referred to one of the responsible researchers who will make an appointment in the operating room for anesthesia (sedation) and closed fracture reduction under fluoroscopic control. Patients who have closed reduction fractures will be randomized using a sealed envelope for one of the two study methods. When the fracture is irreducible, the patient will be excluded from the study. If the fracture reduction is considered inadequate, a second reduction will be made using the same splint construction.

2- Anesthesia - Patients will be subjected to intravenous sedation in the operating room.

3-Immobilization Method- 05 senior hand surgeons will take part in the study, who will supervise previously defined resident physicians and proven to be familiar with both immobilization techniques. The material necessary for the application of both techniques will be available in the operating room. After sedation, the patient will be submitted to closed fracture reduction using the traction and counter-traction technique. Initially, all patients will receive a short radial splint that will be made with a 20 cm wide plaster cut to fit the thumb. The splint will be applied to the radial aspect of the wrist covering the volar and dorsal portion of the radio up to the elbow. Patients randomized for a long splint will receive an immobilization complement with a 15cm wide splint on the ulnar aspect of the forearm that starts at the middle 1/3 of the forearm and goes up to the armpit. The elbow will be immobilized at 90 degrees, neutral to block the prone-supination. Cotton bands and tubular mesh will be used in both immobilizations. Regardless of the immobilization adopted, all wrists will be positioned in slight flexion and ulnar deviation. Patients will be encouraged to actively move their fingers and ipsilateral shoulder.

5- Follow-up:

Both treatment groups will be immobilized for 6 weeks and instructed to return to the clinic in the week following the fracture reduction and in the 2nd, 4th and 6th week after fracture. Patients with AE cast will remain 4 weeks, followed by 2 weeks of short radial immobilization (BE). After removal of the immobilization (6th week), patients will return at 8, 12 and 24 weeks after fracture.

**CLINICAL OUTCOMES**

The self-reported functional assessment (DASH), radiographic, and objective functional assessment will be performed by professionals not directly linked to the study. For outcomes at 8,12 and 24 weeks, the evaluators will be blinded to the patient allocation groups. The minimum clinical follow-up will be 24 weeks, considering the following parameters for the evaluation of results:

1- PRIMARY OUTCOMES:

1a - Self-reported functional outcomes - Functional status will be assessed using the DASH questionnaire at the following times: 2nd, 6th, 8th, 12th and 24th post-fracture weeks. All questionnaires will be supervised by a trained interviewer. The questionnaire was developed by the American Academy and consists of 37 questions related to the function of the hand, wrist, elbow and shoulder. It is validated for the Portuguese language20.

1b - Radiographic parameters - Measurement of angular deviations ^21,22,23^ and associated injuries. The radial height, radial inclination, volar inclination, ulnar variance and articular fragment will be measured, considering the radiographs in the posteroanterior and lateral positions for the evaluation, in the following moments of the treatment evolution: initial, immediate post-reduction (in deviated fractures), 1st, 2nd, 3rd, 4th, 6th, 8th, 12th and 24th week of the fracture. The measurements will be carried out by two researchers independently.

2- SECONDARY OUTCOMES:

2a - Objective functional assessment - Arches of wrist movements for flexion, extension, radial and ulnar deviation will be measured; pronation and supination of the forearm; elbow flexion and extension using a goniometer. Hand grip strength with digital dynamometer (“Jamar Plus - Hand Dynamometer” model), in the following moments of the *treatment evolution: 8, 12 and 24 weeks after fracture.*

2b - Pain - Pain in the wrist, shoulder and elbow will be assessed using the Visual and Analog Scale (VAS) at the following points in the treatment evolution: 1st, 2nd, 3rd, 4th, 6th, 8th, 12th and 24th post-fracture weeks.

2c - PRWE functional questionnaire - Will be evaluated at the 8th, 12th and 24th week after fracture.

2c - Complications- Any clinical situation that requires treatment or clinical or surgical procedure not provided for in the protocol will be considered a complication. All complications will be recorded for further stratification into major and minor complications.

2d - Failure of the method - Any complication that results in interruption or change in the treatment method with the previously randomized method will be considered a failure of the method.

**STATISTICAL ANALYSIS - ANALYSIS BY INTENT TO TREAT**

Epidemiological data will be collected (age, sex, type of fracture, time between fracture and treatment). For such, standard deviations or confidence intervals for percentages will be provided. As a method of confirming the effectiveness of randomization, these data will be compared when stratified by the allocation group.

The verification of the premise of the normality of the distribution will be carried out through the application of the Shapiro-Wilk test, for the use of parametric tests. Pearson's chi-square test will be used to analyze the results of the two groups in relation to the categorical variables. To compare the groups in relation to the numerical variables, the t-student test (parametric) will be used. The paired t-tests (parametric) and Wilcoxon (non-parametric) will be used to compare the clinical evolution at times 2, 3, 4, and 6 weeks after fracture and later at 8, 12 and 24 weeks after fracture. A significance level of 5% (alpha = 0.05) was used in all statistical tests, with tests with a P value less than 0.05 being statistically significant.

If differences are found for primary outcomes, the use of statistical methods is expected to verify whether there is a robust correlation between epidemiological factors or fracture severity with the functional outcomes found. Additionally, it is intended to perform survival analysis and graphic analysis (Survival analysis - Kaplan-Meier curves) if high complication rates (greater than 20%) are found in any of the allocation groups.

Statistical analysis will be performed using Student's T test to compare the maintenance of fracture reduction between the 2 different immobilization groups.

Analysis comparing the percentage of loss of reduction between the two groups in relation to the AO-ASIF classifications.

Student's t test will be used for the analysis of the self-reported function (DASH).

Patients who, for whatever reason, fail treatment or need additional interventions, will be followed up and their results counted in their primary allocation group (principle of intention to treat). Blinding of data for statistical analysis is foreseen by a statistician who does not know the objectives and outcomes of interest.

**RANDOMIZATION AND MASKING**

The decision to include patients in the allocation groups will follow the following randomization method: envelopes will be numbered on their external faces with consecutive numbers, and the method for each envelope will be drawn at random and consecutively using software. randomization (http://www.randomizer.org/). The envelope will only be opened in the operating room after checking the fracture reducibility criteria. The randomization procedure will be carried out by a person not directly linked to the study.

**SAMPLE CALCULATION**

Based on an initial analysis, we calculated that to be able to detect a 10% difference in the arm, shoulder and hand disability questionnaire (DASH) score and anticipating a 10% patient loss in follow-up, a total of 128 distal radius fractures.

**ABBREVIATIONS**

AE: Above-elbow cast; AO: Arbeitsgemeinschaft für Osteosynthesefragen Foundation; ASIF: Association for the Study of Internal Fixation; BE: Below-elbow cast, CEP: Research Ethics Committee; DASH: Disabilities of Arm, Shoulder and Hand; DRF: Distal Radius Fracture, EPM: Escola Paulista de Medicina; VAS: Visual Analog Pain Scale; P: Profile; PA: Posteroanterior; UNIFESP: Federal University of São Paulo.

**REFERENCES**

1-Fernandez DL, Palmer AK. Fractures of the distal radius. In: The Green DP, Hotchkiss RN, Pederson WC, editor(s). Greens Operative Hand Surgery. 4th Edition. New York: Churchill Livingstone: 929-985,1999.

2- Cui Z, Pan J, Yu B, Zhang K, Xiong X. Internal versus external fixation for unstable distal radius fractures: an up-to-date meta-analysis. Int Orthop. 2011 Sep;35(9):1333-41.

3- Diaz-Garcia RJ, Oda T, Shauver MJ, Chung KC. A systematic review of outcomes and complications of treating unstable distal radius fractures in the elderly. J Hand Surg Am. 2011 May;36(5):824-35 e2.

4- Jupiter JB, Fernandez DL. Comparative classification for fractures of the distal end of the radius. J Hand Surg 1997; 22A:563–571.

5- Lichtman DM, Bindra RR, Boyer MI, Putnam MD, Ring D, Slutsky DJ et al. Treatment of distal radius fracture. J Am Acad Orthop Surg 2010;18:180-9.

6- Pool C. Colles’s fracture. A prospective study of treatment. J Bone Joint Surg 1973;55B:540–544.

7- Stewart HD, Innes AR, Burke FD. Functional cast-bracing for Colles’ fractures A comparison between cast-bracing and conventional plaster casts. J Bone Joint Surg 1984;66B:749 – 753.

8- Webb GR, Galpin RD, Armstrongs DG. Comparison of short and long arm plaster casts for displaced fractures in the distal third of the forearm in children. J Bone Joint Surg 2006; 88 A:9-17.

9- Hughston JC. Fractures of the forearm in children. J Bone Joint Surg Am. 1962;44:1678-930

10-Wahlstrom O. Treatment of Colles’ fracture A prospective comparison of three different positions of immobilization. Acta Orthop Scand 1982;53:225–228.

11- Sarmiento A, Pratt GW, Berry NC, Sinclair WF. Colles’ fractures Functional bracing in supination. J Bone Joint Surg 1975;57A:311–317.

12- Bunger C, Solund K, Rasmussen P. Early results after Colles’ fracture: functional bracing in supination vs dor- sal plaster immobilization. Arch Orthop Trauma Surg 1984;103:251–256.

13 - Bong MR, Egol KA, Leibman M, Koval KJ: A comparison of immediate postreduction splinting constructs for controlling initial displacement of fractures of the distal radius: A prospective randomized study of long- arm versus short-arm splinting. *J Hand Surg Am* 2006;31:766-770.

14- Bohm ER, Bubbar V, Hing KY, Dzus A. Above and below-the-elbow plaster cast for distal forearm fractures in children. J Bone J Surg Am. 2006; 88 A:1-8.

15- Hudak PL, Amadio PC, Bombardier C. Development of an upper extremity outcome measure: the DASH (disabilities of the arm, shoulder and hand) [corrected]. The Upper Extremity Collaborative Group (UECG). Am J Ind Med. 1996 Jun;29(6):602-8.

16- Revill SI, Robinson JO, Rosen M, Hogg MIJ. The reliability of a linear analogue for evaluating pain. Anaesthesia. 1976;31(9):1191-8.

17- Rodrigues EKS, Fonseca MCR, MacDermid JC. Brazilian version of yhe patient rated wrist evaluation (PRWE-Br): cross- cultural adaptation, internal consistency, test- retest reliability, and construct validity. J Hand Ther. 2015;28(1):69–76.

18- Müller ME (1996) CCF—Comprehensive Classification of Fractures I & II. M.E.Müller Foundation. Bern: MAO/ASIF Documentation Center.

19- Kreder HJ, Hanel DP, McKee M, Jupiter J, McGilivary G, Swiontowski MF: Consistency of AO fracture classification for the distal radius. J.Bone Joint Surg 78B:726-31,1996.

20 - Orfale AG, Araújo PM, Ferraz MB, Natour J. Translation into Brazilian Portuguese, cultural adaptation and evaluation of the reliability of the disabilities of the arm, shoulder and hand questionnaire. Braz J Med Biol Res. 2005;38:293-302. <https://doi.org/10.1590/S0100-879X2005000200018>

21- Kreder HJ, Hanel DP, McKee M, Jupiter J, McGilivary G, Swiontowski MF: X-ray film measurements for healed distal radius fractures. J Hand Surg [Am] 1996 May;21(3):532.

22- Sharpe F, Stevanovic M. Extra-articular distal radial fracture malunion. Hand Clin. 2005 Aug;21(3):469-87

23- Medoff RJ. Essential radiographic evaluation for distal radius fractures. Hand Clin 2005; 21:279-288.
